# Utility of extracellular vesicles as a potential biological indicator of physiological resilience during military operational stress

**DOI:** 10.14814/phy2.15219

**Published:** 2022-04-04

**Authors:** Meaghan E. Beckner, William R. Conkright, Amrita Sahu, Qi Mi, Zachary J. Clemens, Brian J. Martin, Shawn D. Flanagan, Fabio Ferrarelli, Fabrisia Ambrosio, Bradley C. Nindl

**Affiliations:** ^1^ Neuromuscular Research Laboratory/Warrior Human Performance Research Center Department of Sports Medicine and Nutrition University of Pittsburgh Pittsburgh Pennsylvania USA; ^2^ Department of Physical Medicine & Rehabilitation University of Pittsburgh Pittsburgh Pennsylvania USA; ^3^ School of Medicine University of Pittsburgh Pittsburgh Pennsylvania USA; ^4^ McGowan Institute for Regenerative Medicine University of Pittsburgh Pittsburgh Pennsylvania USA

**Keywords:** decision trees, extracellular vesicles, machine learning, occupational stress, resilience

## Abstract

Extracellular vesicles (EVs) transport biological content between cells to mediate physiological processes. The association between EVs and resilience, the ability to cope with stress, is unknown. Using unbiased machine learning approaches, we aimed to identify a biological profile of resilience. Twenty servicemen (27.8 ± 5.9 years) completed the Connor Davidson Resilience (CD‐RISC) questionnaire and were exposed to daily physical and cognitive exertion with 48‐hr sleep and caloric restriction. Blood samples from baseline and the second day of stress were analyzed for neuroendocrine biomarkers impacted by military stress. EVs were isolated from plasma and stained with antibodies associated with exosomes (CD63), microvesicles (VAMP3), and apoptotic bodies (THSD1). Individuals were separated into high (*n* = 10, CD‐RISC > 90) and low (*n* = 10, CD‐RISC < 79) resilience. EV features were stratified by size, then down‐selected using regression trees and compared between groups. Diagnostic accuracy was assessed using receiver operating characteristic curves. Compared to low resilience, high resilience demonstrated a greater increase in variability of THSD1 local bright spot intensities among large‐sized EVs in response to stress (*p* = 0.002, Hedges’ *g* = 1.59). Among medium‐sized EVs, high resilience exhibited a greater decrease in side scatter intensity (*p* = 0.014, Hedges’ *g* = 1.17). Both features demonstrated high to moderate diagnostic accuracy for high resilience (AUC = 0.90 and 0.79). In contrast, neuroendocrine biomarker concentrations were similar between groups. The increase in variability among THSD1 + EVs in high, but not low, resilient individuals following stress may suggest high resilience is accompanied by stress‐triggered apoptotic adaptations to the environment that are not detected in neuroendocrine biomarkers.

## Graphical abstract

Individuals with high self‐report resilience demonstrated extracellular vesicle adaptations during a multifactorial stress environment that were not observed in low‐resilient individuals. Extracellular vesicles may be more sensitive than circulating neuroendocrine biomarkers, as no differences were observed between groups at the hormone/peptide level.

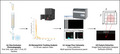

## 1 INTRODUCTION

*Resilience* represents the ability to manage stress and adversity through positive adaptation (Kalisch et al., 2017; Windle et al., 2011). Modern‐day military operations involve physical exertion‐induced fatigue, cognitive overload, and psychological strain, often accompanied by periods of sleep and caloric restriction—making resilience a desirable attribute among military personnel (Lieberman et al., 2016; Nindl et al., 2018). Resilience can be acquired through personal attributes, family dynamics, supportive networks, as well as spiritual and cultural values (Kalisch et al., 2017). As a result, resilience is often considered a trait encompassing an array of characteristics including commitment, self‐efficacy, adaptability to change, and patience, among others (Connor & Davidson, 2003; Liu et al., 2018a). Resilience can also be viewed as a positive physiological stress adaptation when confronted with adversity (Liu et al., 2018a; Nindl et al., 2018). The latter suggests that resilience could also be considered an active process, rather than a static property of personality or trait (Kalisch et al., 2017; Russo et al., 2012).

Self‐report questionnaires are currently one of the most widely used tools to assess trait‐like resilience and have been developed from content themes and constructs of characteristics presumed to embody resilience (Connor & Davidson, 2003). Though there is currently no consensus on the best self‐report measurement of resilience, the Connor–Davidson Resilience Scale (CD‐RISC) (Connor & Davidson, 2003) received among one of the highest ratings in a methodological review of resilience scales based on validity, internal consistency, reproducibility, and interpretability (Windle et al., 2011). The CD‐RISC questionnaire was developed based on the ability to cope with stress by examining acceptance of change, secure relationships, the strengthening effect of stress, personal competence, and sense of control (Connor & Davidson, 2003; Windle et al., 2011). Farina et al. (2019) evaluated CD‐RISC scores among US Army Soldiers enrolled in Special Forces Assessment and Selection (SFAS) and identified that an increase in one standard deviation in CD‐RISC score predicted soldiers were 1.36 times more likely to be selected (Farina et al., 2019). Similarly, Bezdjian et al. (2017) reported that active‐duty enlisted U.S. Air Force Service Members who separated from service within the first 6 months due to unsuitability attrition had significantly lower CD‐RISC scores (76.9 ± 15.5) compared to those who did not (84.1 ± 10.5), with modest discriminability (AUC = 0.64; 95% CI: 0.62–0.65) (Bezdjian et al., 2017).

To examine whether resilience is an *active* process, researchers have turned to biology to identify underlying neurotrophic mechanisms that are linked to resilient outcomes (Russo et al., 2012). Several neuroendocrine biomarkers involved in stress adaptation, including cortisol (Farina et al., 2019; Lieberman et al., 2005; Morgan et al., 2000a; Szivak et al., 2018), neuropeptide‐Y (NPY) (Morgan et al., 2000b; Reichmann & Holzer, 2016), brain‐derived neurotrophic factor (BDNF) (Ledford et al., 2020; Rothman & Mattson, 2013), insulin‐like growth factor‐I (IGF‐I) (Nindl et al., 2007, 2012), and α‐klotho (Prather et al., 2015), have been associated with resilience. Within the past several decades, extracellular vesicles (EVs) have emerged as a pivotal means of cell‐to‐cell communication to aid in regulating normal physiological processes such as tissue repair and immune regulation, as well as the pathology of several diseases (Meldolesi, 2018). Unlike circulating biomarkers that activate a cell through separate signaling pathways, EVs can transfer biological information that can act on multiple targets in a cell simultaneously (Beninson & Fleshner, 2014). EVs are released by nearly all cell types and are comprised of a heterogeneous group of nano‐ to micro‐sized, membrane‐bound vesicles capable of delivering biological content or cargo (i.e., proteins, lipids, nucleic acids) from parent to the recipient cell. EVs are generally categorized based on size and biogenesis into three subpopulations: exosomes, microvesicles, and apoptotic bodies (Akers et al., 2013). Exosomes are formed through a series of invaginations of the cell plasma membrane prior to entering into circulation, ranging in diameter from 30–150 nm (Meldolesi, 2018). Microvesicles are released into circulation via outward budding and fission of the plasma membrane, with varying diameters between 100−1000 nm (Meldolesi, 2018). Apoptotic bodies are larger‐sized EVs (500−5000 nm) formed only during cell death via membrane blebbing, initiated by condensation of nuclear chromatin (Akers et al., 2013; Willms et al., 2018). EVs and their cargo hold promising predictive, diagnostic, and therapeutic capabilities yet to be fully elucidated (Beninson & Fleshner, 2014).

As noted by Battistelli and Falcieri (Battistelli & Falcieri, 2020), each subpopulation of EVs may provide key contributions to intracellular communication involved in health and disease; therefore, it is important that all EV subpopulations are examined. Due to the complexity and diversity of information that can be extracted from EVs, machine learning approaches are often superior to conventional analyses to handle complex multi‐dimensional data and discriminate patterns not easily detected by a few parameters (Sommer & Gerlich, 2013). Thus, the purpose of this study was to identify a biological profile of resilience among individuals with high CD‐RISC scores compared to individuals with low CD‐RISC scores. Machine learning approaches were implemented for an unbiased approach to determine a subset of features among the EV profile, combined with neuroendocrine biomarkers, to compare between groups. We hypothesized that EVs would be a more sensitive indicator of high resilience when compared to neuroendocrine biomarkers at baseline, as well as in response to a controlled stress scenario.

## 2 METHODS

### 2.1 Participants

A subset of participants from a larger study investigating the impact of simulated military operational stress (Beckner et al., 2021) was selected for the present exploratory study of the relationship between biomarkers and resilience. The original cohort was divided into tertiles based on CD‐RISC scores collected at baseline (Beckner et al., 2021). For the present study, ten participants from the top tertile (CD‐RISC Score > 90 out of 100) and 10 participants from the bottom tertile (CD‐RISC score ≤ 79) were randomly selected to compare biological profiles between high and low resilience at baseline and in response to stress.

Participants were recruited through in‐person briefings at local Reserve centers and various website advertisements. Interested individuals contacted the study team to receive further information and complete eligibility screening. Participants were men between the ages of 18 and 41 years currently serving in the U.S. military through Active Duty, Reserve, National Guard, or Reserve Officer Training Corps (ROTC). Eligible participants had no history of sleep, psychotic, or neurological disorders and passed an annual physical fitness test within the last year. An extensive list of inclusion and exclusion criteria has been previously reported (Beckner et al., 2021). The study received approval from the Institutional Review Board at the University of Pittsburgh and the U.S. Army Medical Research and Development Command's Human Research Protection Office (HRPO) and was carried out in accordance with the Declaration of Helsinki. All participants provided written informed consent prior to testing.

### 2.2 Simulated military operational stress protocol

The simulated military operational stress protocol has been described in detail elsewhere (Beckner et al., 2021; Conkright et al., 2021). Briefly, the study protocol was completed over five consecutive days and nights in cohorts of up to four participants at a time. The 5‐day protocol consisted of a familiarization day (D0), baseline day (D1), two days and nights of caloric and sleep restriction (D2 and D3), followed by a final day of testing after a night of uninterrupted sleep (D4). Participants arrived the evening prior to D0 to complete baseline psychological evaluations and one night of familiarization sleep in the accommodations provided by the sleep laboratory. Each day consisted of cognitive testing conducted in a human performance lab, simulated marksmanship on a Reserve installation, and physical exertion in the form of a military obstacle course and ruck march on an indoor turf field. During the restriction phase (D2 and D3), participants were permitted to sleep only 50% of baseline sleep time (from 01:00–03:00 and 05:00–07:00 h) and receive only 50% of their individualized caloric needs, according to sleep polysomnography and estimated energy expenditure using whole‐body densitometry (Bod Pod^®^ Body Composition System, Life Measurement Instruments, Concord, CA), respectively. On all other days, participants were permitted to sleep from 11:00–07:00 h and allotted their full caloric needs. Water was permitted *ad libitum* throughout the study.

### 2.3 Psychological measures

The CD‐RISC scale is a 25‐item self‐assessment that uses a 5‐point scale (0–4) to measure resilience based on previously identified characteristics shared among resilient people. Total scores range from 0 to 100 with higher scores being associated with higher resilience (Connor & Davidson, 2003). The CD‐RISC has good internal consistency (Cronbach's α = 0.89), test‐retest reliability (ICC = 0.87), and convergent validity with the Perceived Stress Scale (*r* = −0.76, *p* < 0.001) (Connor & Davidson, 2003). Therefore, we interpreted resilience as the ability to cope with stress in accordance with the CD‐RISC questionnaire developed by Connor and Davidson (Connor & Davidson, 2003).

To better characterize psychological differences between high and low resilient individuals, combat experience, coping strategies, and personality traits were also assessed. Combat experience was evaluated using a subscale of the Deployment Risk and Resilience Inventory‐2 (DDRI‐2) that included 17 objective combat‐related circumstances (Vogt et al., 2012). Participants were instructed to indicate how often they experienced each circumstance on a scale of 1–6 (i.e., *never* to *daily or almost daily*). Total scores ranged from 17 to 102 with higher scores indicating greater combat exposure. The Coping Styles and Strategies (COPE) 60‐item scale assesses 15 types of strategies to manage and react to adverse or unexpected situations: Positive reinterpretation, active coping, planning, seeking of social support for emotional reasons, seeking of social support for instrumental reasons, suppression of competing activities, religion, acceptance, mental disengagement, focus on and venting of emotions, behavioral disengagement, denial, restraint coping, alcohol/drug use, and humor (Carver et al., 1989). The questionnaire contains four questions per coping strategy. Participants were instructed to rate how they handle stressful events on a scale from one to four (i.e., *I don't do this at all* to *I usually do this a lot*) (Carver et al., 1989). Ratings for each strategy were then totaled for a minimum score of four (*not at all*) to 16 (*does a lot*). Personality was assessed using the NEO Five‐Factor Inventory‐3 (NEO‐FFI‐3^TM^), a 60‐item questionnaire that measures the five domains of personality: Neuroticism, extraversion, openness, agreeableness, and conscientiousness (McCrae & Costa, 2010). Individuals rate each statement on a 5‐point Likert scale from 0 to 4 (i.e., *disagree strongly* to *agree strongly*). The 12 items for each domain were summed, ranging from 0 to 48, with higher scores indicating higher levels of that personality trait.

Participants completed all questionnaires after signing consent and prior to any physical testing or stress intervention.

### 2.4 Biological specimens

Fasted blood was drawn from an upper extremity vein using a 21‐ or 23‐gauge needle (BD Vacutainer Eclipse 22 g and Vacutainer one‐use holder, Becton, Dickinson, and Company, Franklin Lakes, NJ) the first morning (~08:00) of the 5‐day protocol as a baseline assessment (i.e., Day 0), and repeated the morning after two consecutive nights of sleep restriction, considered the peak stress day (i.e., Day 3). Using standard venipuncture procedures, 10 ml of serum in an SST tube, 10 ml of blood plasma in an EDTA collection tube, and 2 ml in EDTA with a protease inhibitor (BD^TM^ P100) (BD Vacutainer Becton, Dickinson, and Company, Franklin Lakes, NJ), were collected. EDTA and protease inhibitor tubes were centrifuged immediately after collection at 1500 ×* g* for 15 min at 4°C. SST tube was centrifuged under the same conditions following 30 min at room temperature to allow blood to clot. The supernatant was aliquoted and stored at −80°C until further analysis.

ELISA assays were used to measure IGF‐I (APLCO, Salem, USA) and α‐Klotho (Immuno‐Biological Laboratories, Takasaki, Japan) using plasma samples from EDTA collection tubes. BDNF was analyzed from blood plasma using MILLIPLEX Magnetic Bead Panel 3 (EMD Millipore, Burlington, Massachusetts). Plasma obtained from EDTA tubes with a protease inhibitor was used for NPY analysis (R&D Systems, Minneapolis, MN, USA). Cortisol was analyzed using serum samples (Alpco Salem, USA). Kit sensitivity is as follows: IGF‐I: 0.09 ng/ml; α‐Klotho: 6.15 pg/ml; BDNF: 10 pg/ml; cortisol: 0.4 µg/dl. This information was not available for NPY. All samples were run in duplicate with intra‐assay coefficients of variation of 10% or less.

### 2.5 Size exclusion chromatography

**FIGURE 1:**
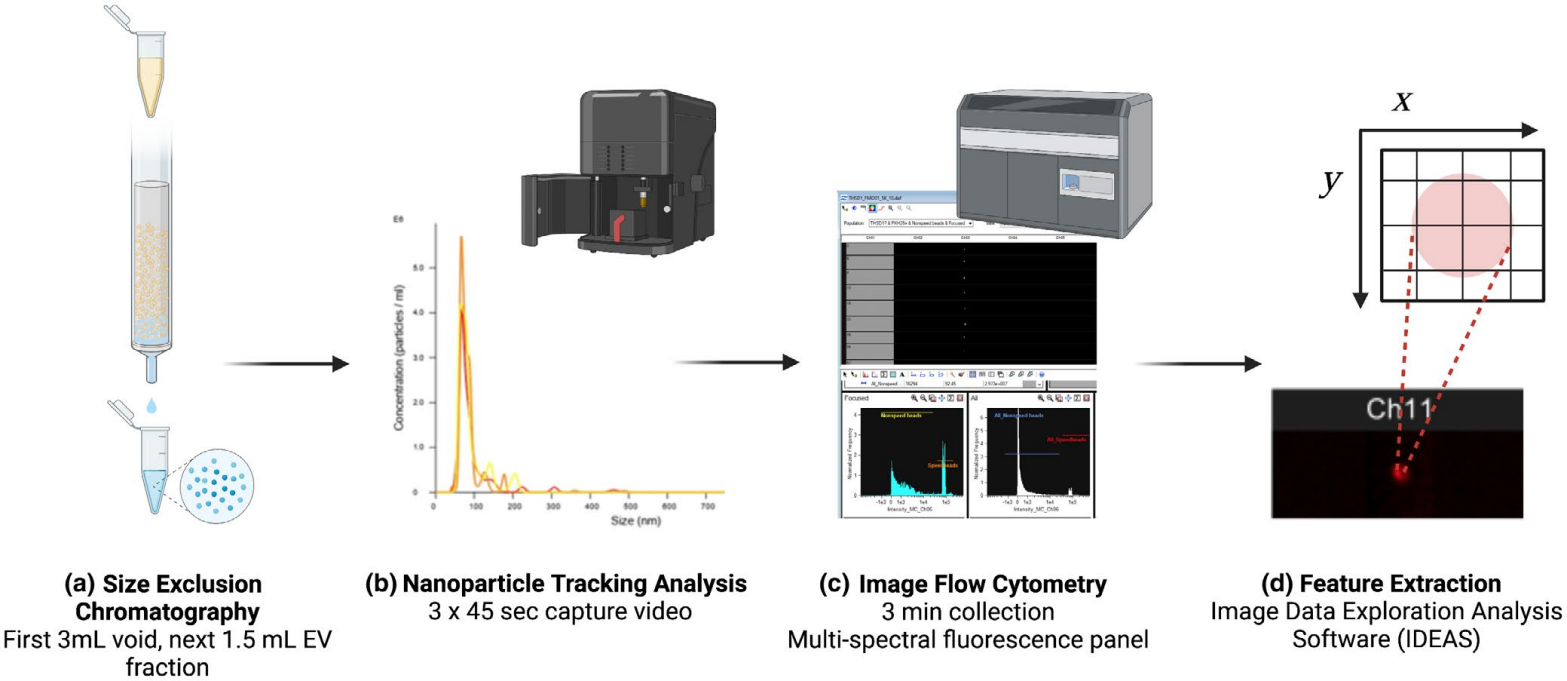
Overview of extracellular vesicle (EV) analysis. (a) EVs were isolated from plasma samples using size exclusion chromatography (SEC). (b) EV concentrations and size were measured using nanoparticle tracking analysis (NTA). (c) EV samples were stained with immunofluorescence markers associated with exosomes (CD63), microvesicles (VAMP3), and apoptotic bodies (THSD1) and then assessed using imagine flow cytometry to collect multispectral images of each EV. (d) Quantitative information from the images, known as features, were exported for analysis. Figure created with BioRender.com

A visual overview of the EV analysis process is depicted in Figure 1. First, EVs were isolated from plasma samples (ETDA collection tubes) using 70 nm size exclusion chromatography (SEC) columns, per manufacturer's instructions (qEVoriginal, Izon, Medford, MA). Plasma samples were brought to room temperature and centrifuged at 1500 ×* g* for 10 min. SEC columns were first flushed with 10 ml of 0.22 µm filtered EV‐free phosphate‐buffered saline (PBS) solution, after which 450 µl of the plasma sample was loaded into the column, and fractions were collected as they eluted. The first 3 ml of eluate was discarded, and the subsequent 1.5 ml EV fraction was collected in a microcentrifuge tube. The following 4.5 ml after the EV fraction, primarily plasma protein eluate, was discarded. Columns were flushed with 15 ml PBS between samples, with the same column used for up to five samples. Isolated EV samples were stored at 4°C and EV size and concentration were analyzed within 48 h, after which time EV samples were frozen until subsequent analysis.

### 2.6 Nanoparticle tracking analysis

Nanoparticle tracking analysis (NTA) is a commonly used method to measure EV concentration and size distribution, based on light scattering from particles under Brownian motion, captured by a charge‐coupled device camera (CCD) (Dragovic et al., 2011). EV concentration and size were analyzed using NS300 NanoSight device equipped with a green laser (Malvern Panalytical). Ten microliters of isolated EVs were diluted 1:100 in Type 1 EV‐free water and loaded into the sample chamber using a syringe. Total EV size and average concentration were derived from 3 × 45 s video captures with the camera set to Level 14 (NTA 3.4 Build 3.4.003). The flow‐cell was washed with 1 ml of Type 1 water between each sample.

### 2.7 Immunofluorescence staining of EV subpopulations for imaging flow cytometry

Frozen EV samples were thawed at room temperature, vortexed, and 140 µl from each sample was placed into a new Eppendorf tube and fixed with an equal volume of 4% paraformaldehyde solution. Samples were incubated at room temperature for 10 min, then centrifuged at 16,000 ×* g* at 4ºC for 30 min (Thermo Scientific Fiberlite F21‐48 × 1.5/2/.0 rotor). Afterwards, 140 µl of supernatant was extracted and discarded from each sample and 140 µl of blocking buffer (3% bovine serum albumin and 0.1% Triton‐X) was added. Samples were placed on a rocker plate and incubated at room temperature for 1 h, then centrifuged at 16,000 ×* g* for 30 min at 4ºC, after which 140 µl of supernatant was removed and discarded. EV samples were then stained with fluorescently conjugated antibodies associated with EV subpopulation surface markers as follows (Akers et al., 2013): Exosomes, CD63 (Novus Biologicals, NBP2‐42225AF700) 1:280 dilution; microvesicles, VAMP3 (Novus Biologicals, NBP1‐97948AF405) 1:280 dilution; and apoptotic bodies, THSD1 (Novus Biologicals, FAB5178T‐100UG) 1:1000 dilution. Following an overnight incubation in the dark at 4ºC, samples were centrifuged at 16,000 ×* g* for 30 min at 4ºC and 60 µl of supernatant was removed and discarded. EVs were resuspended with 20 µl of PBS and analyzed using imaging flow cytometry. Compensation beads (Invitrogen, UltraComp eBeads, 01‐2222‐42) for each EV marker were also stained following the same procedure, beginning with blocking buffer and using half the dilution for antibody staining, in order to apply fluorescence compensation for analysis.

Imaging flow cytometry (IFC) combines flow cytometry and single‐cell imaging, to capture up to 12 spatially registered multi‐spectral images per cell as it passes through the system (Gorgens et al., 2019; Hennig et al., 2017; Lannigan & Erdbruegger, 2017). The 60x objective, longer signal integration times, and slower flow rates with IFC lead to increased sensitivity for characterization of EVs compared to conventional flow cytometry (Lannigan & Erdbruegger, 2017). EV samples were imaged on ImageStream^X^ Mk II system (Luminex Corporation, Seattle, WA) at the flow cytometry core of the Department of Immunology at the University of Pittsburgh. Fluorescently labeled EVs in solution were run through the ImageStream^X^ Mk II and data were acquired using the INSPIRE control software. Laser settings were set to maximum intensity, magnification set to 60x, and fluidics set to low speed/high sensitivity with a core size of 7 µm for optimal detection of nano‐sized vesicles. SpeedBeads^®^ were removed from event detection by plotting a histogram of side‐scatter (SSC) intensity and removing events with >1e + 5 SSC intensity. All samples were acquired for a run time of 3 min. Compensation beads for each antibody were collected until a threshold of 2000 events was met. The INSPIRE acquisition software generated data in the form of raw image files for all samples and controls.

### 2.8 Image data exploration and analysis software

Image Data Exploration and Analysis Software (IDEAS) is the most common analysis software for IFC and IDEAS allows the user to employ a range of features derived from each event detected (Hennig et al., 2017). A spectral compensation of all antibody stained control image files was applied to sample images to correct for variances in‐camera background, flow speed, and fluorescence compensation by subtracting light emitted by fluorochromes in the neighboring channel (IDEAS, 2020). The IDEAS software extracts over 100 features, or quantitative information about the image, that are categorized based on size, shape, texture, and signal strength. All sample image files with feature values were exported as a.csv file for statistical analysis (IDEAS, 2020).

### 2.9 IDEAS data reduction

Individual.csv files for each sample were reduced to remove 6 of the 12 spectral‐image channels that did not correspond to the fluorochromes used for this study. Three redundant features were removed, and five features were removed as they were deemed irrelevant to the research question. An object (i.e., EV) with a Saturation Count ≥1 in the fluorescence channels was removed as possible debris or fluorochrome aggregates A new feature was generated for each object to capture the range of pixel intensities within an object, calculated as the Raw Max Pixel minus the Raw Min Pixel for each object.

To further examine the heterogeneity within each EV sample, objects detected were stratified by the area of the brightfield image to capture changes that may occur in specific EV size ranges. “Small” EVs were categorized as objects with a brightfield image area <0.031416 µm^2^, “medium” EVs with an area ≥0.031416 µm^2^ but ≤0.785398 µm^2^, and “large” EVs > 0.785398 µm^2^. Size stratification cut‐offs were derived from diameters typically used in EV literature to characterize exosomes, microvesicles, and apoptotic bodies, respectively (Willms et al., 2018). The addition of size stratification allowed the use of both size and antibody to distinguish EV subpopulations. Descriptive statistics (i.e., mean, median, and standard deviation) were then calculated for every feature at each stratum as well as for the total sample (i.e., without stratification) to account for the overlap in size ranges of EV subpopulations. The data reduction process yielded a total of four variables per feature (i.e., small, medium, large, and total EVs) for each of the three antibodies (i.e., CD63, VAMP3, and THSD1). For example, the mean intensity feature was determined for small EVs/CD63+, mediums EVs/CD63+, large EVs/CD63+, and all EVs/CD63+. The same four variables were determined for the mean intensity of VAMP3+ and THSD1+ EVs. Therefore, 12 variables were generated for a given feature, totaling 1116 variables per sample.

### 2.10 Feature selection using machine learning

Considering that 1116 unique features were generated from each EV sample, regression tree (RT) models were implemented as a data mining methodology for feature selection to identify the most salient input variables for statistical analyses to characterize resilience. The RT model is a binary decision tree that uses variable selection to identify subgroups of a population and ultimately generate homogenous terminal nodes in relation to the dependent variable (Machuca et al., 2017; Mendeş & Akkartal, 2009). The decision tree begins with a root node containing all subjects which are then split into two mutually exclusive subsets based on an independent variable, followed by internal nodes (i.e., subsequent subdivisions of the subset based on other independent variables), and ending with terminal nodes or subsets that can no longer be split due to homogeneity or due to stopping criteria to avoid the model from becoming overly complex (Mendeş & Akkartal, 2009; Song & Lu, 2015).

**FIGURE 2:**
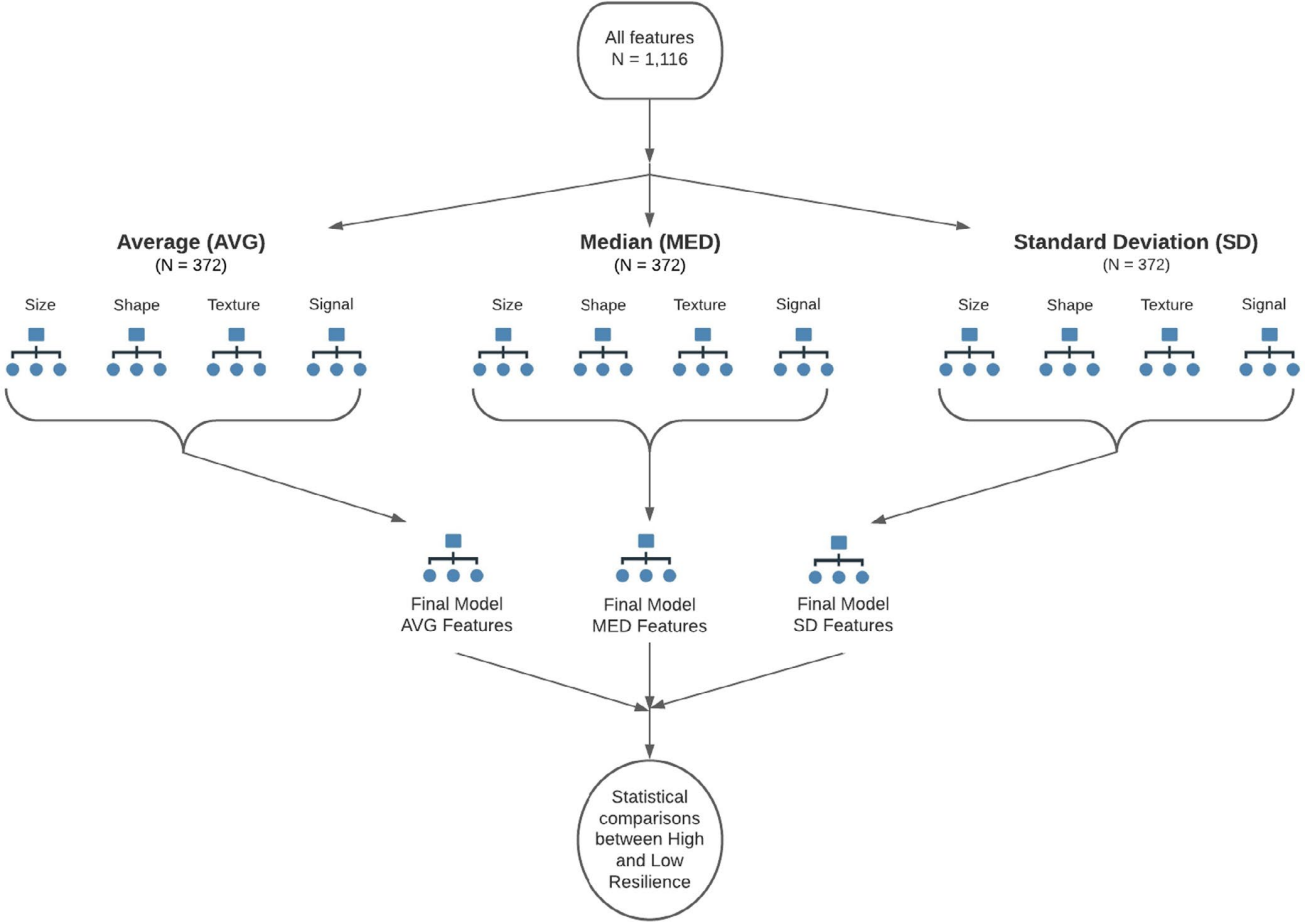
Extracellular feature selection using regression decision trees. Four regression tree (RT) models based on feature category were generated based on the average, median, and standard deviation of features. The features that were identified in each RT model at the first level for a given statistic (i.e., average, median, or standard deviation) were then used as input variables for a final RT model for each statistic at the second level. The EV features identified from each of the three final RT models were analyzed using statistical comparisons to determine differences between high and low resilient individuals

The risk estimate value is an estimation of the within‐node variance for a continuous dependent variable (i.e., CD‐RISC score) (Mendeş & Akkartal, 2009). The independent variable (i.e., EV feature) used to split a node is determined by the Least Squared Deviation (LSD) method to measure the variance within a node (Mendeş & Akkartal, 2009). The independent variable with the lowest risk estimate value is the best splitter variable as it produces the lowest within node variance. The unit of the risk estimate value is based on the dependent variable unit and was normalized by dividing the risk estimate value by the variance of the dependent variable (Mendeş & Akkartal, 2009). The RT workflow for EV feature selection is depicted in Figure 2. All regression trees were obtained using the Classification Regression Tree (CRT) model in IBM SPSS Statistics for Macintosh, Version 27 (IBM Corp., Armonk, NY).

For assessing a biological profile of resilience at baseline, EV samples from D0 were used. Data reduction of the ImageStream features generated 372 features based on the average (AVG) of all events within a sample, 372 features based on the median (MED) of all events within a sample, and 372 features based on the standard deviation (SD) of all events within a sample to capture the variability of events within the sample. Features were categorized into four categories as defined in the IDEAS User Manual Version 6.3 (IDEAS, 2020): Size (96 features), shape (12 features), texture (72 features), and signal strength (192 features). For each statistic (i.e., AVG, MED, SD), RTs were made for each feature category with CD‐RISC score as the continuous dependent variable to identify the most discriminatory EV features of resilience within each subtype. Due to the small total number of observations (*N* = 20), the following stopping rules were implemented: (a) A minimum of five observations (i.e., 25% of the total sample) in a terminal node, (b) minimum of 10 observations in a node prior to splitting, and (c) a maximum tree depth of three levels. The features identified from the four feature subtype RTs were then used as the independent variables in an RT model to identify the most discriminatory features of resilience across all feature subtypes for a given statistic. This process was repeated for each statistic to generate three final RT models: One for AVG, one for MED, and one for SD. The features identified in the three final RT models were then used as the input variables for statistical analysis comparing differences in the EV profile at baseline between high and low resilient individuals based on CD‐RISC Score.

For assessing a biological profile of resilience based on the stress response, absolute change scores were calculated to assess the change in EV profile from baseline to peak stress by subtracting feature values of the D0 EV sample from the feature values of the D3 EV sample (i.e., D3–D0). The same RT model workflow previously described was followed using the change scores to identify feature changes able to discriminate between high and low resilience, which were then subsequently used as the input variables for statistical analysis.

### 2.11 Statistical analysis

Normality of distribution for the independent variables was assessed using Shapiro–Wilk tests. The p‐value was set at 0.05 (two‐sided), *a priori*, for all analyses. To examine the overall impact of the stressor from baseline to peak stress, neuroendocrine responses, and EV concentration were assessed using paired samples *t*‐test or Wilcoxon signed rank test, as appropriate. To compare baseline and change scores of neuroendocrine biomarkers and EV features between high and low resilience, independent samples *t*‐test were used for normally distributed variables and the Mann–Whitney *U* test was used with an exact sampling distribution for *U* for non‐normal variables. Hedges’ *g* values were calculated for significant outcomes from independent samples *t*‐test to measure the magnitude of the difference between groups. Data analyzed using non‐parametric statistics are reported as median (MED) and interquartile range (IQR). Figures are displayed as mean with standard deviation. Outcomes with a significant difference between groups were evaluated using receiver operating characteristic (ROC) curve analysis to determine the diagnostic accuracy of the variable to discriminate between high resilient and low resilient individuals as determined by CD‐RISC score. The area under the curve (AUC) with 95% *CI* was calculated to determine the overall diagnostic accuracy of the variable, with an AUC of 0.5 indicating random chance and AUC = 1 indicating perfect discrimination (Hajian‐Tilaki, 2013). Likelihood ratio (LR), the likelihood a positive result will be identified in a person with high resilience compared to a person with low resilience, was also calculated. A LR > 10 is considered a large conclusive change, a LR between 5 and 10 is regarded as moderate, and an LR < 2 is seldomly recognized as important or a valuable diagnostic test (Hajian‐Tilaki, 2013). The optimal cut point of the curve was determined by the maximum value of the Youden index (*J*) for each variable (Hajian‐Tilaki, 2013). All statistical measures were obtained using IBM SPSS Statistics for Macintosh, Version 27 (IBM Corp., Armonk, NY); ROC curves and analyses were obtained using GraphPad Prism, Version 9.1.1 (GraphPad Software LLC, La Jolla, CA).

### 2.12 Steps to ensure rigor

To reduce bias, investigators performing data acquisition including ELISA assays, EV isolation, immunofluorescence staining, and imaging flow cytometry were blinded to group allocation (i.e., high/low resilience). All sample conditions (i.e., D0 and D3) for a given subject and an equal number of high and low resilient samples were analyzed on the same day. An investigator not involved with data acquisition selected and coded the appropriate samples for analysis each day.

## 3 RESULTS

### 3.1 Baseline characteristics and impact of simulated military operational stress

High resilient individuals had an average CD‐RISC score of 94.90 ± 3.04 out of a maximum score of 100 whereas the average score of low resilient individuals was 70.00 ± 5.89. The demographic information for the sample included in this study is provided in Table 1. The two groups were similar in age, combat experience, years of service, aerobic fitness, and body composition. Low resilient individuals were slightly taller than high resilient individuals [*p* = 0.02, Hedges’ *g* = 1.09 (95% CI 0.16, 1.99)]. Individuals with high resilience reported engaging in coping strategies pertaining to positive reinterpretation (*p* < 0.001), active coping (*p* = 0.007), planning (*p* < 0.001), seeking of instrumental social support (*p* = 0.01, Hedges’ *g* = 2.68), and suppression of competing activities (*p* = 0.002, Hedges’ *g* = 2.01) to a greater extent than low resilient individuals. No other significant differences in coping strategies were observed. High resilient individuals also demonstrated significantly higher levels of conscientiousness (i.e., self‐discipline, *p* < 0.001, Hedges’ *g* = 4.36) and extraversion (i.e., outgoing, *p* < 0.001) than low resilient individuals. Conversely, low resilient individuals demonstrated significantly higher levels of neuroticism, or the tendency to experience negative emotion (*p* < 0.001, Hedges’ *g* = 5.68).

**TABLE 1:** Participant characteristics (*N* = 20)

	Low resilience (*n* = 10)	High resilience (*n* = 10)	All (*N* = 20)
Age (years)	28.13 ± 5.81	27.47 ± 6.22	27.80 ± 5.87
Height (cm)^a^	181.13 ± 5.76	174.05 ± 6.67	177.59 ± 7.07
Weight (kg)	92.46 ± 18.80	80.45 ± 14.63	86.45 ± 17.50
BMI (kg/m^2^)	28.20 ± 5.71	26.51 ± 4.40	27.36 ± 5.03
Body fat (%)	23.26 ± 7.18	20.24 ± 6.70	21.75 ± 6.93
VO_2peak_ (ml∙kg∙min^−1^)	47.73 ± 7.04	47.66 ± 11.04	47.69 ± 9.00
CD‐RISC score^a^	70.00 ± 5.89	94.90 ± 3.04	82.45 ± 13.56
DRRI‐2 score	21.80 ± 7.12	23.00 ± 13.02	22.40 ± 10.23
Total years of service	7.08 ± 6.23	8.73 ± 6.15	7.90 ± 6.08

Note

Data presented as mean ± standard deviation.

Abbreviations: BMI, body mass index; CD‐RISC, Connor Davidson resilience scale; DDRI‐2, deployment risk and resilience inventory‐2.

^a^ Significant difference between groups (*p* < 0.05).

At baseline, participants consumed 2378.4 ± 420.2 kcal∙d^−1^ (32% fat, 56% carbohydrate, 12% protein) and slept 7.3 ± 0.4 h. In contrast, participants consumed on average 1447.5 ± 194.4 kcal∙d^−1^ (29% fat, 60% carbohydrate, 11% protein) and slept 3.8 ± 0.2 during the stress scenario (i.e., days 2 and 3).

### 3.2 Neuroendocrine biomarkers

To assess if concentrations of circulating neuroendocrine biomarkers were differentially expressed in high and low resilient individuals, we analyzed α‐Klotho, BDNF, NPY, IGF‐I, and cortisol at the onset of the 5 d SMOS protocol as a baseline measure as well as the second consecutive day of sleep and caloric restriction, considered the peak stress of the 5 d protocol. No group differences were observed for neuroendocrine biomarkers at baseline between high and low resilience groups (Table 2).

**TABLE 2:** Baseline neuroendocrine concentrations

	Low resilience (*n* = 10)	High resilience (*n* = 10)
α‐Klotho (pg/ml)		
Mean ± SD	1013.69 ± 332.89	956.37 ± 341.04
Median [IQR]	852.02 [527.03]	871.19 [542.84]
BDNF (pg/ml)		
Mean ± SD	5595.60 ± 6548.55	6273.00 ± 4592.35
Median [IQR]	2638.00 [5778.75]	4917.50 [6567.75]
NPY (pg/ml)		
Mean ± SD	2210.53 ± 993.55	3594.32 ± 2496.45
Median [IQR]	1782.01 [1751.00]	2684.44 [4718.89]
IGF‐I (ng/ml)		
Mean ± SD	273.57 ± 64.37	293.05 ± 91.62
Median [IQR]	278.49 [112.41]	276.77 [98.44]
Cortisol (µg/dl)		
Mean ± SD	26.15 ± 11.63	28.21 ± 9.57
Median [IQR]	22.23 [20.40]	23.84 [12.99]

Note

Independent samples *t*‐test or Mann–Whitney *U* test (as appropriate) indicated there were no significant differences between high and low resilient individuals at baseline.

Abbreviations: BDNF, brain‐derived neurotrophic factor; IGF‐I, insulin‐like growth factor I; NPY, neuropeptide‐Y.

**FIGURE 3:**
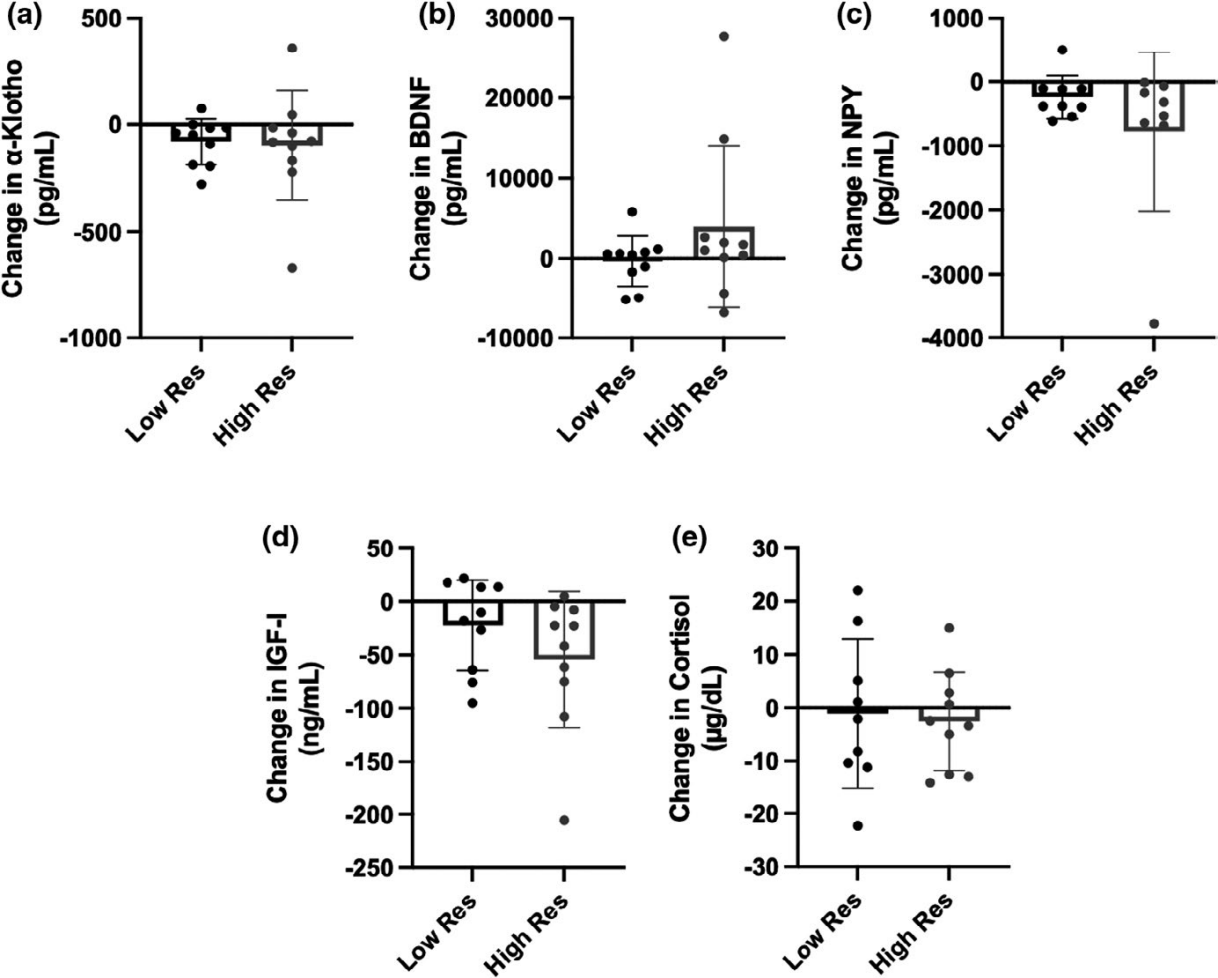
Changes in neuroendocrine biomarker concentrations from baseline to peak stress between high and low resilient (Res) individuals. Absolute change scores were compared between low resilient (*n* = 10) and high resilient (*n* = 10) individuals using independent samples *t*‐test or Mann‐Whitney U test, as appropriate. No significant differences in response to stress were observed in (a) α‐Klotho, (b) brain‐derived neurotrophic factor (BDNF), (c) neuropeptide‐Y (NPY), (d) insulin‐like growth factor I (IGF‐I), or (e) cortisol. Data are presented as mean and standard deviation of the change score

Overall, the 48 h of restricted sleep and caloric intake elicited significant declines in α‐Klotho (*p* = 0.01), IGF‐I (*p* = 0.006, Hedges’ *g* = 0.681), and NPY (*p* = 0.002) among all participants. No significant changes were observed in BDNF or cortisol from baseline to peak stress. Similar changes in biomarker concentrations were observed between groups, with no significant differences between high and low resilience (Figure 3).

### 3.3 EV characterization

**FIGURE 4:**
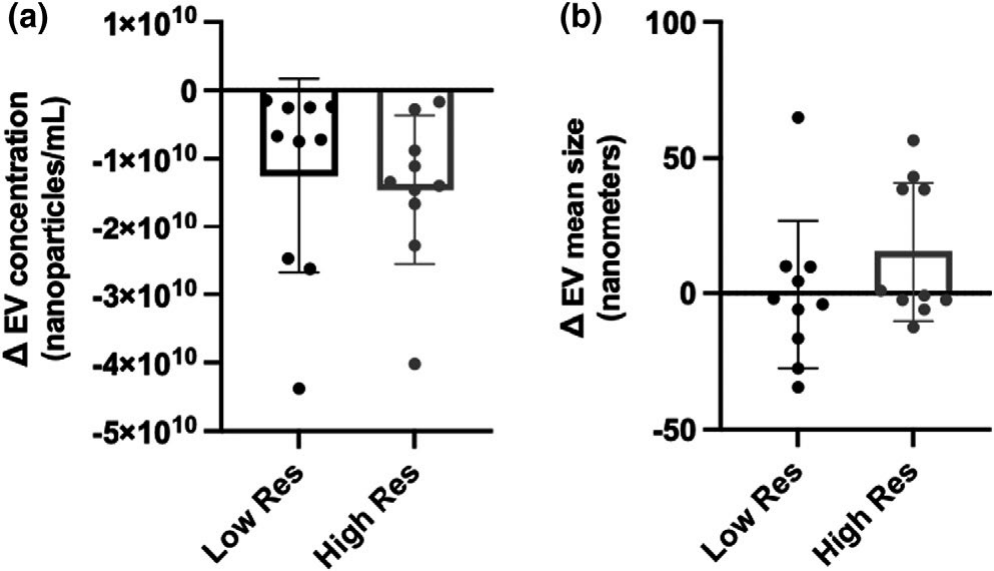
Changes in extracellular vesicle (EV) characterization from baseline to peak stress. Absolute change scores were compared between low resilient (*n* = 10) and high resilient (*n* = 10) individuals using independent samples *t*‐test or Mann‐Whitney U test, as appropriate. No significant differences were observed in response to stress between low and high resilient (Res) individuals in (a) EV concentration or (b) EV mean size

EV concentration and mean size were similar between high and low resilient individuals at baseline (*p* = 0.823 and *p* = 0.148, respectively) (Table 3). Overall, there was no significant change from baseline to peak stress in EV concentration (*p* = 0.222) or EV size (*p* = 0.575). Likewise, there was no significant difference in change scores for EV concentration (*p* = 0.353) or average EV size (*p* = 0.205) between high and low resilience (Figure 4).

**TABLE 3:** Baseline EV characterization

	Low resilience (*n* = 10)	High resilience (*n* = 10)
Concentration (×10^10^ nanoparticles/ml)		
Mean ± SD	2.78 ± 1.81	2.60 ± 1.67
Median [IQR]	2.95 [3.00]	2.12 [2.00]
Mean size (nanometers)		
Mean ± SD	112.63 ± 24.86	97.68 ± 18.96
Median [IQR]	102.55 [46.15]	96.05 [20.03]

Note

Independent samples *t*‐test or Mann–Whitney *U* test (as appropriate) indicated there were no significant differences in EV characterization between high and low resilient individuals at baseline. Data presented as mean ± standard deviation (SD) and median with interquartile range [IQR].

### 3.4 Feature selection based on EV features at baseline

Next, we sought to take an unbiased approach to select baseline EV features that were best able to discriminate between high and low resilient individuals using regression tree (RT) models, based on the average, median, and standard deviation of EV features. Separate RT models for the mean, median, and standard deviation of EV features at baseline, as well as the final RT model, are presented in the Supplemental Material (Figures S1–S4). Ultimately, the regression tree models selected five features of interest to decipher resilience: (a) The average and (b) median raw maximum pixel intensity of brightfield image in large‐sized EVs, (c) the average intensity of THSD1+ EVs among medium‐sized EVs, (d) the median bright detail intensity of THSD1+ among large‐sized EVs, and (e) the variability of side scatter aspect ratio. We subsequently analyzed these five features to determine if there was a significant difference between high and low resilient individuals for each feature.

### 3.5 Comparison of baseline EV features between high and low resilient individuals

**FIGURE 5:**
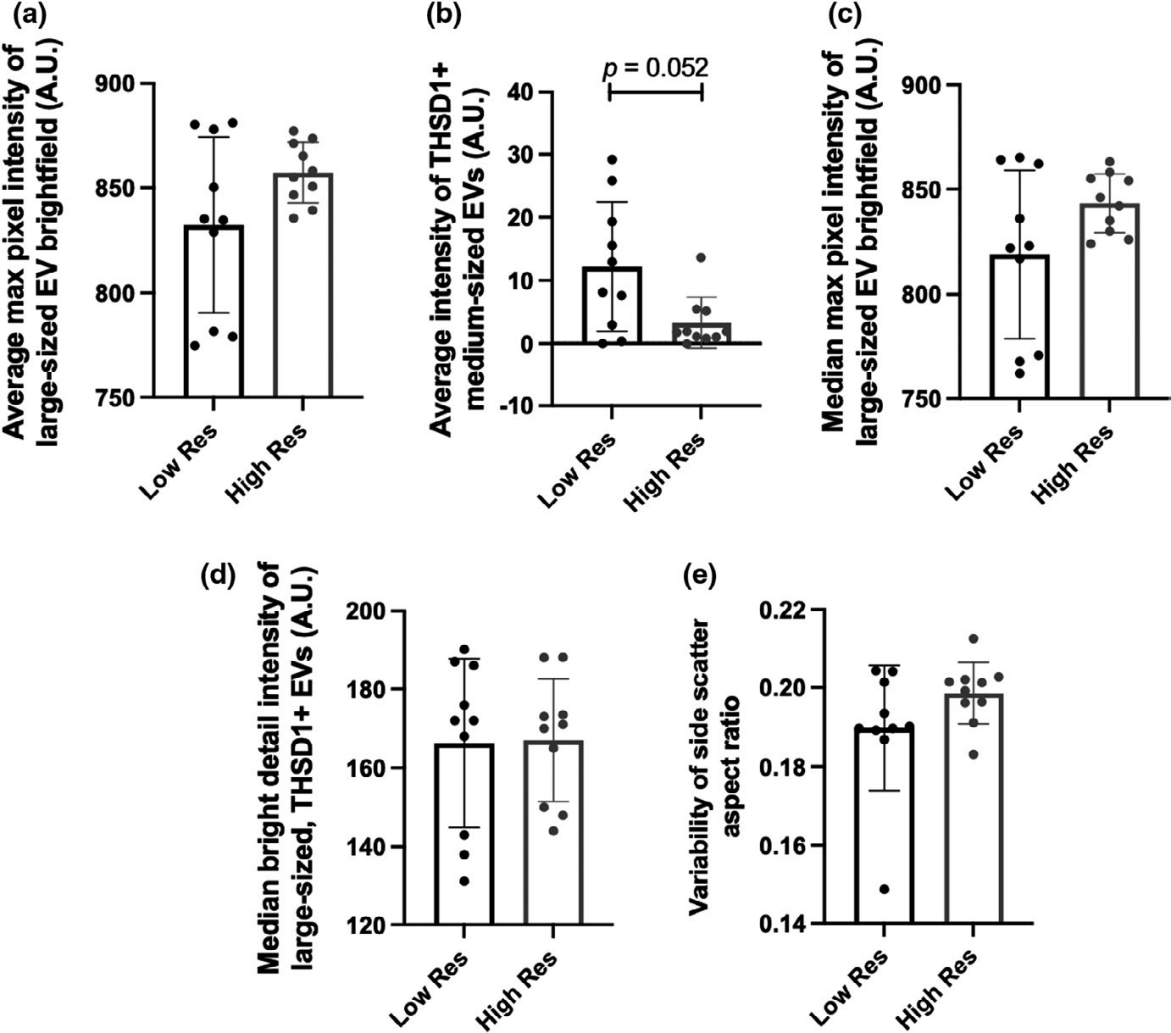
Comparison of EV features at baseline between low and high resilient (Res) individuals. Independent samples *t*‐test or Mann‐Whitney U test (as appropriate) were used to compare differences in EV features at baseline between low resilient (*n* = 10) and high resilient (*n* = 10) individuals. No significant differences were observed at baseline in (a) the average maximum pixel intensity in brightfield images of large‐sized EVs, (b) the average intensity among medium‐sized THSD1+ EVs, (c) the median maximum pixel intensity in brightfield images of large‐sized EVs, (d) the median intensity of localized THSD1+ bright spots (defined as bright sports in the image that are 3 pixels in radius or less) among large‐sized EVs, and (e) the variability of side scatter aspect ratio, a measure of circularity, with an aspect ratio of 1 indicating a perfect circle

Four of the five baseline EV features were non‐normally distributed and analyzed using the Mann–Whitney *U* test to compare between high and low resilience. Independent samples *t*‐test was used to assess the median bright detail intensity of THSD1 among large‐sized EVs, as this variable was normally distributed. Similar to baseline neuroendocrine markers, no significant group differences were identified among the EV features at baseline (Figure 5). However, the average intensity of THSD1 among medium‐sized EVs approached significance (*p* = 0.052), with low resilient individuals having the greater average intensity of THSD1 among medium‐sized EVs (MED: 10.54; IQR: 2.32, 20.95) compared to high resilient individuals (MED: 3.29; IQR: 0.97, 5.24).

### 3.6 Feature selection based on EV feature changes in response to stress

Provided that many definitions of resilience rest on how an individual responds to a stressor, we investigated whether changes in the EV profile in response to 48 h of sleep and caloric restriction would differ between those that are highly resilient compared to low resilient peers. We repeated the same feature selection decision tree process as executed with baseline EV features, except EV feature absolute change scores were used as the independent variables. Individual RT models for the mean change, median change, and standard deviation of change for EV features in response to stress, as well as the final RT model using absolute change scores, are presented in the Supplemental Material (Figures S5–S8). The regression tree models determined four features of interest in discriminating resilience based on changes in EV features in response to stress: (a) Average change in EV side scatter minimum pixel intensity, specifically within medium‐sized EVs; (b) average change in maximum pixel intensity of THSD1, specifically within large‐sized EVs; (c) median change in area of the brightfield image of all EVs; and (d) the variability of the change in bright detail intensity of THSD1+ large‐sized EVs. These four features were then analyzed to determine which features displayed significant differences between high and low resilient individuals.

### 3.7 Comparison of EV feature changes in response to stress between high and low resilient soldiers

**FIGURE 6:**
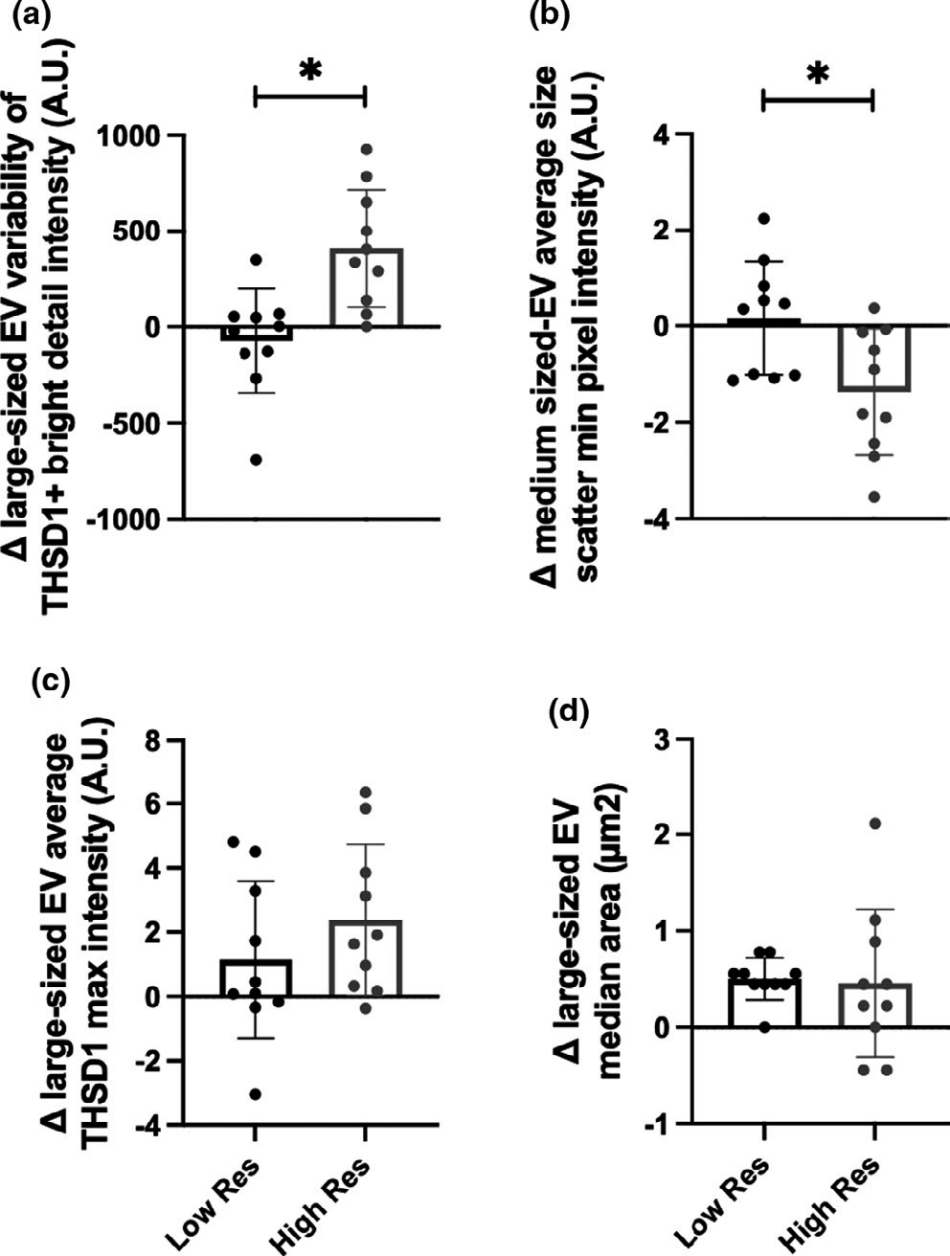
Comparison of EV feature changes in response to stress between low and high resilient (Res) individuals. Absolute change scores were compared between low resilient (*n* = 10) and high resilient (*n* = 10) individuals using independent samples *t*‐test or Mann‐Whitney U test, as appropriate. A significant (*p < 0.05) difference between high and low resilient individuals in response to the stressor were observed in (a) the change in intensity variability of localized THSD1+ bright spots among large‐sized EVs and (b) the change in average minimum side scatter intensity among medium‐sized EVs. No significant differences were observed in response to stress in (c) average maximum pixel intensity of large‐sized THSD1+ EVs or (d) the median area in brightfield images of large‐sized EVs

While no differences were observed between groups when comparing circulating neuroendocrine biomarker changes in response to stress, select EV features responded differently to stress when individuals were stratified according to resilience score (Figure 6). Three of the four EV feature change scores were normally distributed and, thus, analyzed using an independent samples *t*‐test. The median change in the area of the brightfield image among large‐sized EVs was analyzed using the Mann–Whitney *U* test due to violations of normality. Most notably, the variability of the change in bright detail intensity of large‐sized THSD1+ EVs was significantly greater among high resilient individuals compared to low resilient individuals [*p* = 0.002, Hedges’ *g *= 1.59 (95% CI: 0.59, 2.56)] (Figure 6a). In contrast, the average change in minimum side scatter pixel value among medium‐sized EVs decreased in high resilient individuals in response to stress compared to the minimum change observed in low resilient individuals [*p* = 0.014, Hedges’ *g *= 1.17 (95% CI: 0.12, 2.16)] (Figure 6b). No significant differences were observed between groups in the average change of maximum pixel intensity of large‐sized THSD1+ EVs (*p* = 0.262) or the median change in the area of the brightfield image of all EVs (*p* = 0.446).

### 3.8 Characteristic performance of EV features

**FIGURE 7:**
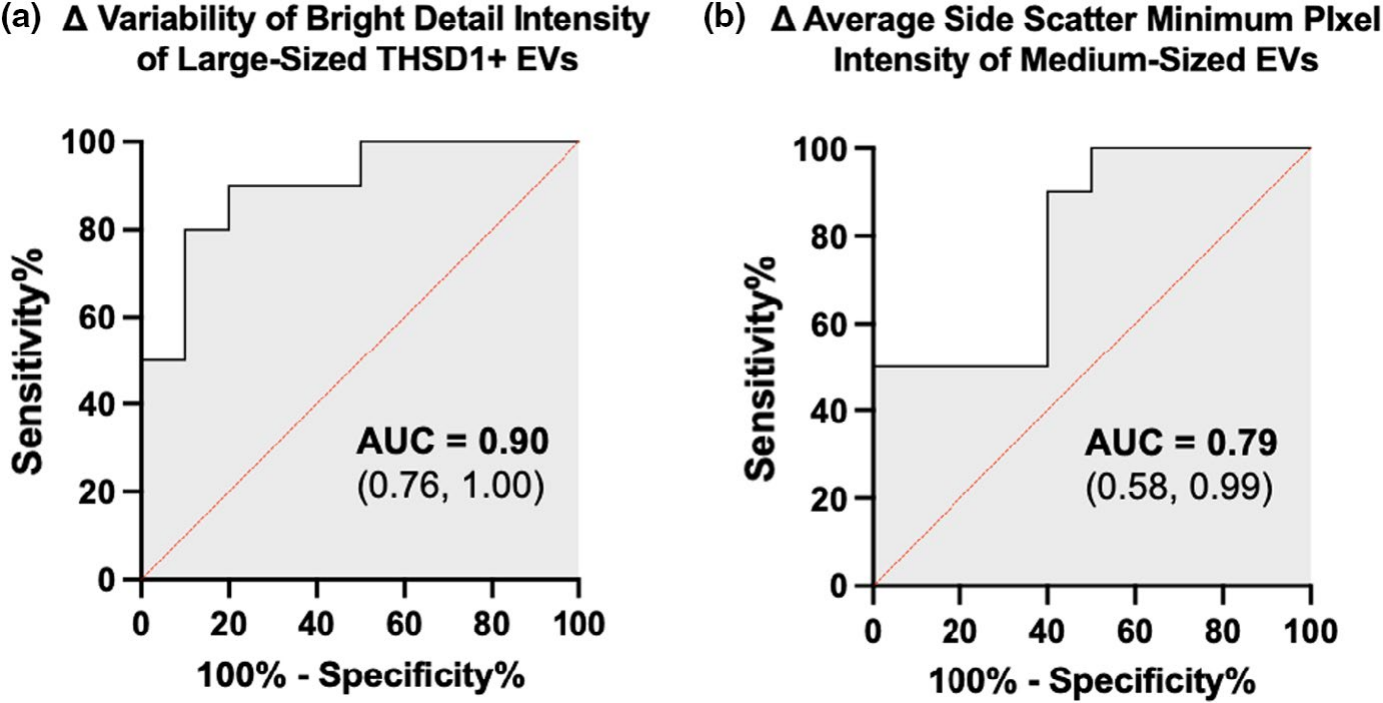
Receiver‐operating characteristic (ROC) curve depicting the ability of EV features to characterize resilience among soldiers. (a) the change in intensity variability of localized THSD1+ bright spots among large‐sized EVs (b) the change in average minimum side scatter intensity among medium‐sized EVs. AUC, area under the curve; (95% Confidence Interval)

To determine how well the variability of the change in bright detail intensity of THSD1+ large‐sized EVs and the average change in minimum side scatter pixel value among medium‐sized EVs distinguished high from low resilient individuals, we plotted ROC curves based on the rule used to classify individuals as being highly resilient. For variability of the change in bright detail intensity of THSD1+ large‐sized EVs, the area under the ROC curve was 0.90 (95% CI: 0.76–1.00, *p* = 0.003) (Figure 7a). In this sample population, the optimal cutoff as determined by the Youden index (*J*) was a change score standard deviation of >101.00 with 80% sensitivity (95% CI: 49.02–96.45%) and 90% specificity (95% CI: 59.58%–99.49%), yielding an 8.00 likelihood ratio (LH). The ROC curve for the average change in minimum side scatter pixel value among medium‐sized EVs displayed an AUC of 0.79 (95% CI: 0.58–0.99, *p* = 0.028) with an optimal cut off at an average change <0.140 corresponding to 90% sensitivity (95% CI: 59.58–99.49%) and 60% specificity (95% CI: 31.27–83.18%) with a 2.25 LH (Figure 7b).

## 4 DISCUSSION

The purpose of this investigation was to identify a biological profile able to discriminate highly resilient individuals from low resilient counterparts based on CD‐RISC scores. Contrary to our hypothesis, none of the neuroendocrine biomarkers or EV features were able to discriminate highversus low resilience at baseline. However, the results supported our hypothesis that EVs were a more sensitive indicator of high resilience compared to neuroendocrine biomarkers in response to a controlled stress scenario. While changes in neuroendocrine biomarkers were unable to differentiate resilience, we observed changes within EV features following 48 h of sleep and caloric restriction that were significantly different between high and low resilient individuals. Specifically, the variability of change in bright spot intensities of large‐sized THSD1+ EVs, a marker associated with apoptotic bodies, following the restriction period was significantly greater among high resilient individuals. Additionally, highly resilient individuals exhibited a decrease in a minimum side scatter pixel intensity, a measure of cell complexity. Low resilient individuals demonstrated little to no change in EV features following the 48 h sleep and caloric restriction period. The responses observed were likely a reflection of cumulative stress, rather than acute stress, as blood was obtained at rest on both baseline and peak stress days.

### 4.1 High trait‐resilience is characterized by greater heterogeneity among EVs associated with apoptotic bodies, which may be beneficial for adaptation during stress

The average CD‐RISC score of the cohort in the present study (82.4 ± 13.7) mirrors that of other military populations, namely U.S. Navy SEAL candidates (Ledford et al., 2020) and active duty U.S. Air Force service members (Bezdjian et al., 2017), which are above mean scores reported among U.S. college students, ranging from 67.7 to 70.6 (Steinhardt & Dolbier, 2008). Interviews conducted with individuals presumed to embody resilience, including Navy SEALs and children of the Great Depression, have revealed common core characteristics among resilient individuals to include calm‐thinking, decisive action, tenacity, and a positive perspective on life (Everly et al., 2012). Our results also demonstrated that high resilient individuals were more likely to use positive reinterpretation, active coping, planning, seeking of social support, and suppression of competing activities to cope with stress—all of which are characterized as problem‐focused coping strategies aimed at taking action to alter the source of the stress (Carver et al., 1989). Certainly, these are key attributes in a military environment where remaining calm under pressure can be critical to mission readiness and the difference between life or death.

Individuals with CD‐RISC scores >90 in the present study demonstrated greater heterogeneity among large‐sized THSD1+ EVs, associated with apoptotic bodies, in response to the multi‐factorial stress scenario. THSD1 is part of a family of thrombospondin extracellular proteins involved in cell‐to‐cell and cell‐to‐matrix communication, regulating cellular processes from tissue genesis and repair to cell attachment, motility, and proliferation (Friedl et al., 2002). THSD1 acts as a molecular bridge between phagocytic and apoptotic cells and plays a key role in the recognition and phagocytosis of cells undergoing apoptosis, or programmed cell death (Friedl et al., 2002). Apoptotic bodies, which are formed by the breakdown of nuclear chromatin, are the largest‐sized subpopulation of EVs (Akers et al., 2013). Our results demonstrated that increased heterogeneity of THSD1+ EVs in high resilient individuals was specific to large‐sized EVs, providing additional support that these changes were occurring within the apoptotic body subpopulation. Viable cells contain numerous intracellular structures and are complex, producing high amounts of side scatter with flow cytometry as the photon strikes the inner contents; however, as cells fragment into apoptotic bodies, side scatter decreases due to less photon obstruction (Jiang et al., 2016). The lower minimum side scatter intensity among high resilient individuals in response to stress further endorse an increase in apoptotic bodies observed in those with high CD‐RISC scores.

Apoptotic bodies were once regarded as nothing more than cellular debris. However, they are now known to carry a considerable amount of RNA relative to other EV subpopulations that can be engulfed by macrophages and prime molecular memory through the transfer of intercellular contents (Battistelli & Falcieri, 2020; Liu et al., 2018b). Furthermore, the lipid membrane of apoptotic bodies precludes the inner contents from being released into the surroundings, preventing an inflammatory reaction (Battistelli & Falcieri, 2020). The protein composition of plasma apoptotic cells in healthy humans are associated with various biological processes including cellular component organization, biogenesis, metabolism, and response to stimuli, among others (Serrano‐Heras et al., 2020). It is hypothesized that acute stress‐triggered apoptosis may be beneficial for adaptations to the environment as it can cause physiological changes in the brain, generating new neurons and increasing plasticity (McKernan et al., 2009). Therefore, the increase in variability THSD1+ EVs in high resilient individuals, but not low resilient individuals, following 48 h multi‐factorial stress may suggest high resilience is accompanied by stress‐triggered apoptotic adaptations to the environment.

The beneficial role of apoptotic bodies has been investigated in animal models by observing the physiological impact of an absence of apoptotic bodies. Liu et al. (2018b) observed that apoptosis‐deficient mice, characterized by Fas deficiency and caspase three knockouts, had significantly reduced apoptotic body formation accompanied by impaired self‐renewal and differentiation of bone marrow mesenchymal cells. However, when the apoptosis‐deficient mice were infused with exogenous apoptotic bodies weekly for 4 weeks, mesenchymal cells were restored and the osteopenia phenotype was mitigated (Liu et al., 2018b). Similarly, apoptotic bodies were shown to functionally modulate liver macrophage homeostasis to counteract Type 2 diabetes, improving glucose tolerance and insulin sensitivity (Zheng et al., 2021). Apoptotic bodies are enveloped and ingested by macrophages, dendritic cells, and endothelial cells, then degraded by lysosomes (Liu et al., 2018b). The proteomic and transcriptomic components within apoptotic bodies are conjectured to facilitate molecular memory and the transfer of cellular information via the engulfment and degradation process (Liu et al., 2018b). Of note, excessive rates of apoptosis can lead to pathology (Liu et al., 2018b); therefore, optimal rates of apoptosis under specific environmental conditions need to be further investigated.

### 4.2 Neuroendocrine biomarkers were unable to distinguish high trait‐resilience

While we observed significant declines in IGF‐I (–13.5%), α‐Klotho (–8.9%), and NPY (–17.2%) from baseline to peak stress among all participants, there was no difference in neuroendocrine response between high‐ and low‐resilient individuals. The observed decreases in IGF‐I concentrations are consistent with previous reports demonstrating IGF‐I concentrations are attenuated during rigorous military training (Nindl et al., 2007, 2012). Likewise, high‐stressed individuals have been reported to have lower concentrations of α‐Klotho (Prather et al., 2015). Previous military studies have demonstrated increases in NPY concentrations immediately following mock captivity (Morgan et al., 2000b; Szivak et al., 2018). However, Szivak et al. (2018) demonstrated NPY concentrations significantly declined 24‐hr following completion of military survival training compared to baseline. In the present study, blood was collected in the morning upon awakening, not following acute stress, which may have contributed to the directional changes observed in NPY. Despite the fact that no changes in BDNF concentrations were observed in the present investigation, the parent study demonstrated declines in BDNF were more apparent at the onset of stress, rather than at peak stress (Beckner et al., 2021). Contrary to numerous studies reporting significant increases in cortisol during military training scenarios (Lieberman et al., 2016; Morgan et al., 2000a; Szivak et al., 2018), we observed no changes in cortisol concentrations from baseline to peak stress. Considering the baseline blood was collected approximately ~14 h after the onset of the study protocol due to one night of familiarization sleep in the sleep laboratory, it is plausible participants’ cortisol may have already been elevated at the time of baseline blood collection. In support of this, cortisol concentration at the baseline time point in the present study (~26–28 µg/dl) was more similar to average concentrations following mock interrogations than baseline concentrations in other military stressors (Lieberman et al., 2016; Morgan et al., 2000a; Szivak et al., 2018).

EVs present several advantages over circulating neuroendocrine biomarkers that may have contributed to the changes observed among EVs, but not circulating biomarkers, in response to the 48 h sleep and caloric restriction. Unlike circulating biomarkers, EVs are a comprehensive package of biological content that is protected from degradation in circulation by the EV’s lipid bilayer (Beninson & Fleshner, 2014). Furthermore, the EV lipid bilayer increases the stability of biological content during extended exposure to room temperature or freeze and thaw cycles (if stored below −70°C), which makes it a desirable biomarker in field studies that often encounter unexpected delays in sample processing or analysis (Hackl et al., 2016).

EV cargo includes nucleic acids and proteins, as well as non‐coding RNA such as microRNA (miRNA), that mirror the biological content of the parent cell and regulate gene expression post‐transcription (Akers et al., 2013; Keifer et al., 2015; Meldolesi, 2018). Exposure to physiological challenges or stressors has been reported to modify miRNA, which is packaged in a targeted manner rather than coincidental incorporation into EVs, that act on hundreds of messenger RNA and modifies protein expression (Beninson & Fleshner, 2014). Provided that resilience is a multi‐factorial phenomenon, EVs may provide a more individualized biological signature of resilience that may be masked at the hormone/peptide level due to a lack of sensitivity and specificity (Schmidt et al., 2011). Though the biological content of EVs was not investigated in the present study, consideration in future studies is warranted to understand the relationship between resilience and EV cargo.

### 4.3 Unbiased selection confirmed by diagnostic accuracy support EVs as a candidate biomarker of resilience

The utilization of imaging flow cytometry for EV analysis provided a high‐throughput measurement of morphometric features at the single‐EV level. Due to the abundance of information collected, feature selection was an important first step in the analysis as more features do not necessarily improve the performance of a diagnostic or classification algorithm (Sommer & Gerlich, 2013). For example, Loo et al. (2007) demonstrated that of approximately 300 single‐cell phenotypic features, only ~20 features enhanced the interpretability of drug response and detection of phenotypic changes in the cells, with little compromise in classification accuracy. Here, we utilized machine learning‐based RT models for an *unbiased* approach to down select EV features most capable of classifying resilience. The RT model used in this study is a non‐parametric method that does not require assumptions about the distribution of the independent variables, is not affected by multicollinearity, and can be used on small datasets (Mendeş & Akkartal, 2009). Furthermore, the RT model can simplify complicated relationships between the independent variables and the dependent variable by splitting the sample into subgroups based on select independent variables (Song & Lu, 2015).

The EV features down‐selected by the RT model based on resilience scores were supported by subsequent diagnostic accuracy testing. Circulating biomarkers that reflect biological pathways activated during a stressful scenario, such as the HPA axis, have been associated with favorable military performance and, presumably, resilience (Morgan et al., 2001). However, a statistically significant association (*p* < 0.05) between biomarkers and the outcome is not sufficient to determine the diagnostic or predictive value (Wang, 2011). Discrimination, the ability to distinguish those with the outcome of interest versus those that do not, is an important criterion when considering the utility of a biomarker (Wang, 2011). The change in EV features related to apoptotic bodies in the present study was not only significantly different between high and low resilient individuals, but the AUCs demonstrated both features exhibited moderate to high discrimination accuracy.

### 4.4 Considerations

There is not currently a consensus for defining resilience in the scientific literature and objective quantification is elusive. Resilience is a phenomenon which is largely inferred, primarily through self‐report questionnaires, leading to variability in how it is defined, operationalized, and measured (Kalisch et al., 2017; Windle et al., 2011). Self‐report assessments depend on the individual's knowledge of the objective truth, ability to recognize the truth, and willingness to report it (Lazarus, 2000). Demand characteristics, the tendency for a subject being evaluated to alter responses or behaviors in a way that is perceived as favorable, can lead to inflated scores on self‐assessments of personality constructs, such as resilience (Bartone, 2006). However, studies by Farina et al. (2019) and Bezdjian et al. (2017) have demonstrated the utility of self‐report resilience in that settings. Additionally, this study was exploratory in nature and conducted with a small sample of 10 highly resilient and 10 low‐resilient individuals from a larger study (Beckner et al., 2021). Therefore, these results should be interpreted with caution and confirmed in a larger sample of individuals covering a broad spectrum of resilience scores and military experience. It is important to note that the blood measures in this study were obtained at rest rather than immediately following an acute stressor, which may have yielded a different response pattern. Rather, resting blood measures collected on the third day reflect the cumulative stress of two consecutive nights of sleep restriction and reduced caloric intake. Lastly, this study aimed to examine differences in the overall heterogeneity of the EV subpopulation between high and low resilience, using one surface marker protein per subpopulation. Including measurements of the nucleic and/or proteomic material carried by EVs is warranted in future studies and would enrich our knowledge of EVs with respect to resilience and stress tolerance.

## 5 CONCLUSION

Whether resilience is a trait or a process remains largely debated in the literature (Liu et al., 2018b). Our results suggest that trait‐like resilience (based on CD‐RISC score) is accompanied by a physiological process, as demonstrated by EV adaptations in response to stress observed in highly resilient individuals, but not observed in low resilient individuals (Liu et al., 2018b). Similar to physiological adaptations that occur with strength and aerobic training (Friedl et al., 2015), physiological adaptations may occur as a result of repeated environmental exposures, altering cognitive appraisal, which may contribute to enhancing resilience (Kalisch et al., 2015; Yao & Hsieh, 2019). Furthermore, the future of EVs shows promise for more sensitive diagnostic power and the capability for engineered EVs to be potential therapeutic interventions for various diseases (Dou et al., 2020; Man et al., 2020), which could be the future of enhancing resilience.

## CONFLICT OF INTEREST

No conflicts of interest, financial or otherwise, are declared by the author(s).

## AUTHOR CONTRIBUTION

BCN, FA, and MEB conceived and designed research; MEB, WRC, AS, and ZJC performed experiments; MEB, WRC, and QM analyzed the data; MEB, WRC, FA, AS, QM interpreted results of experiments; MEB and WRC prepared figures; MEB drafted manuscript; FA, BCN, QM, WRC, and FF edited and revised manuscript; MEB, WRC, QM, AS, ZJC, BJM, SDF, FF, FA, and BCN approved final version of the manuscript.

## Supporting information



Fig S1
