# Supplementary material for: Utility of extracellular vesicles as a potential biological indicator of physiological resilience during military operational stress
Source: Physiol Rep. 2022 Apr 4;10(7):e15219. doi: 10.14814/phy2.15219 (PMC8978596; doi:10.14814/phy2.15219)
Supplement: Supplementary file 1 — Fig S1 [file PHY2-10-e15219-s001.docx]

**SUPPLEMENTAL DATA**


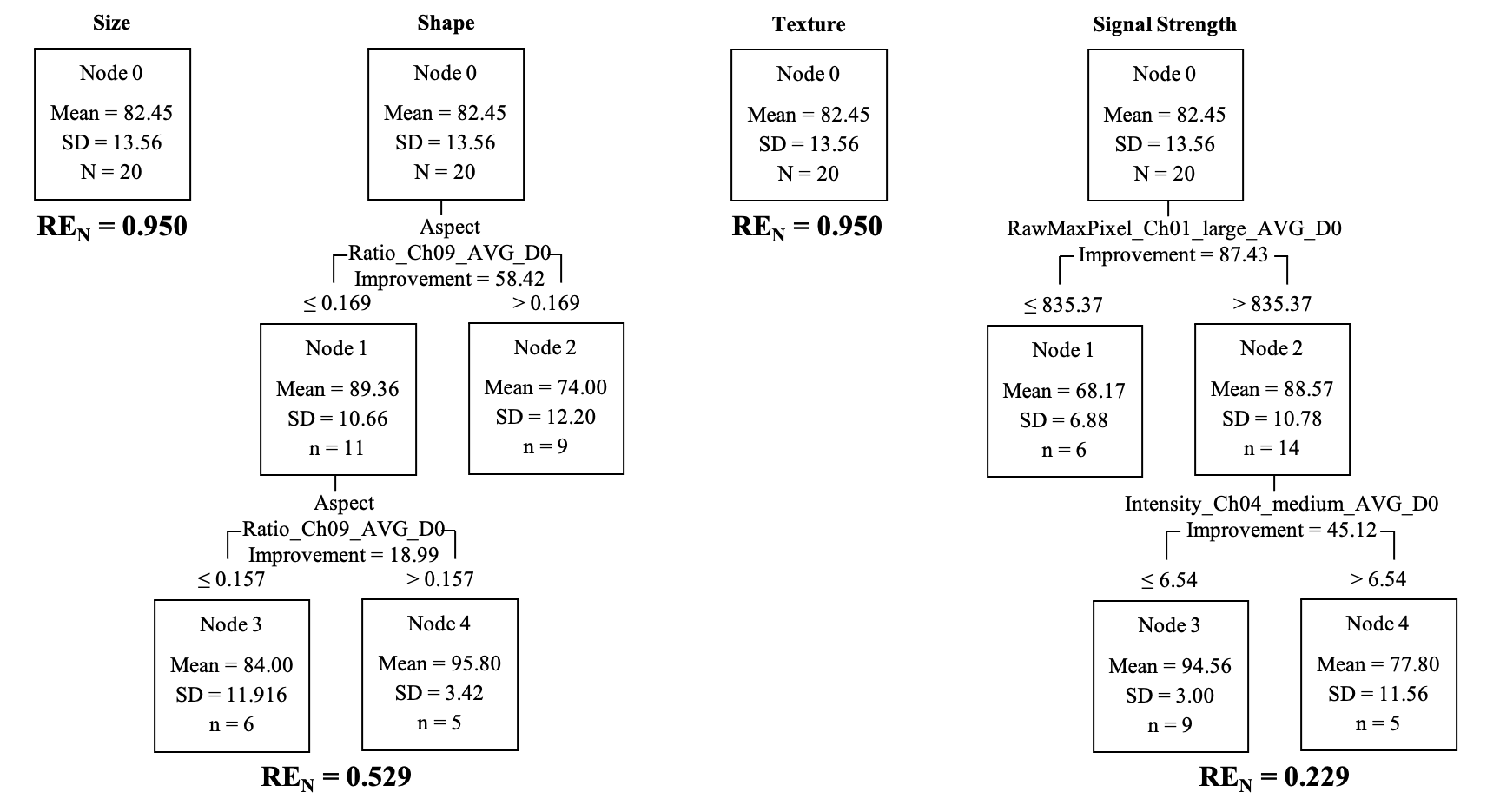


**Figure S1. Regression tree of resilience scores using baseline average (AVG) EV features.** Two features within the shape category and one feature of signal strength were able to discriminate resilience; no features among the size of texture category did not generate child nodes. RE_N_ = normalized risk estimate.


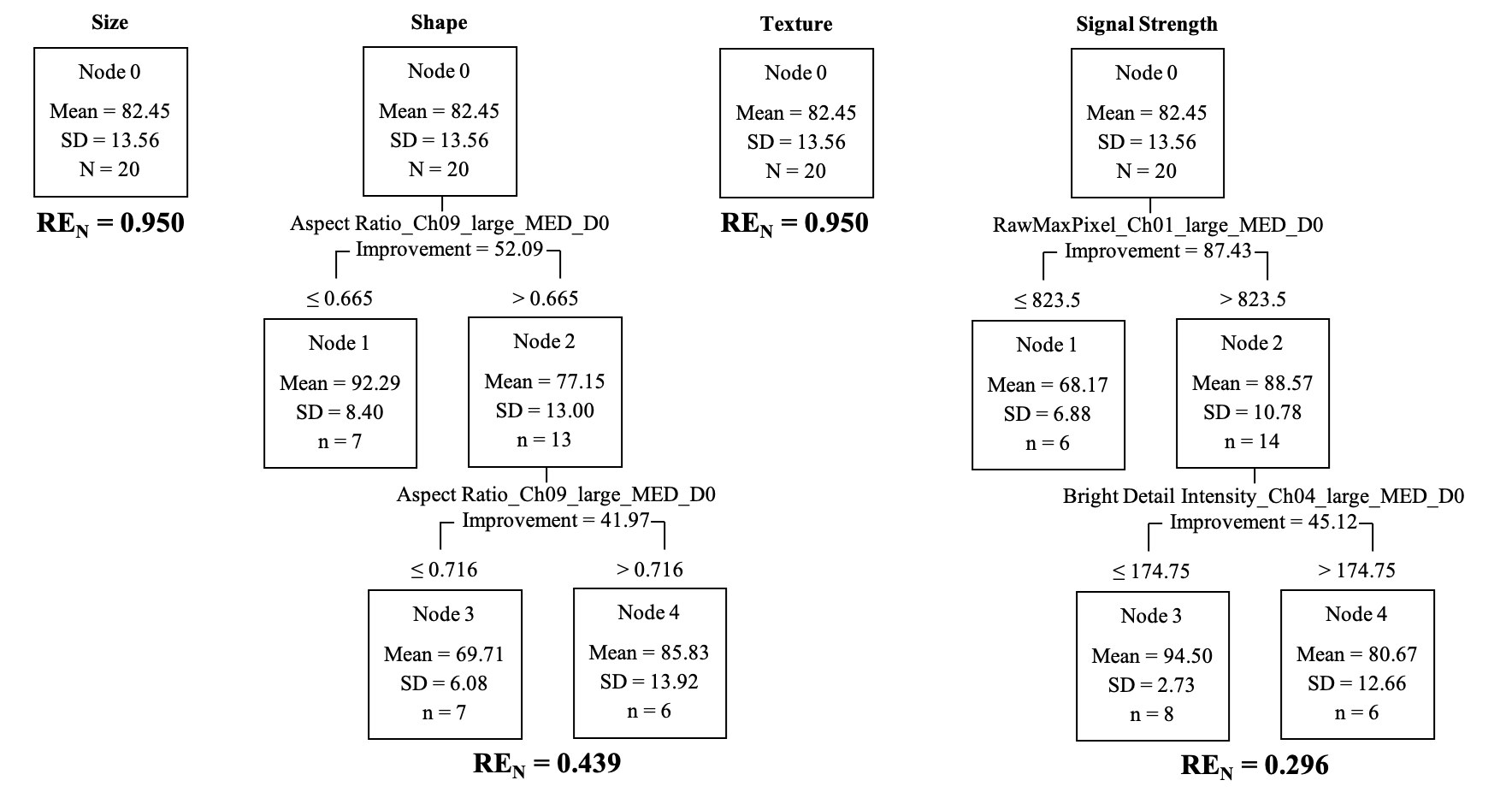


**Figure S2. Regression tree of resilience scores using baseline median (MED) EV features**. Two features within the shape category and two features of signal strength were able to discriminate resilience; no features among the size of texture category did not generate child nodes. RE_N_ = normalized risk estimate. RE_N_ = normalized risk estimate.


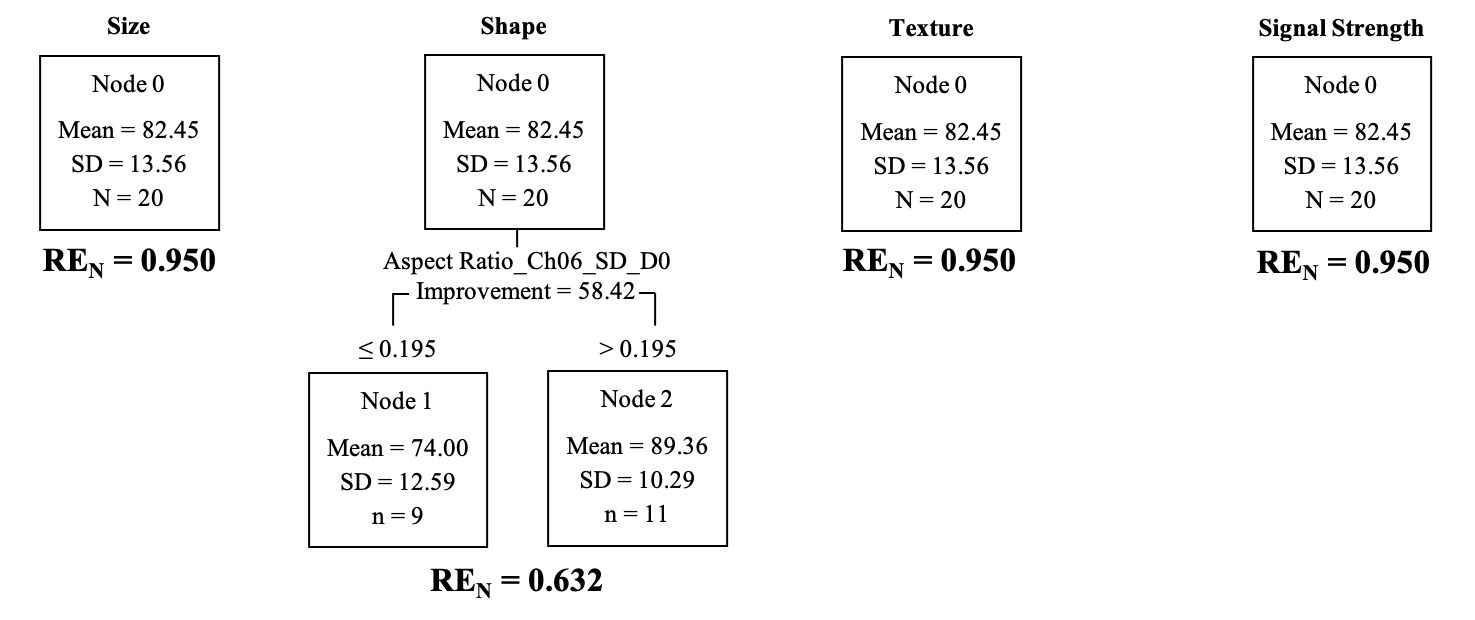


**Figure S3. Regression tree of resilience scores using baseline standard deviation (SD) EV features**. Only one shape feature was identified among the four decision tree models based on EV feature variability. RE_N_ = normalized risk estimate.


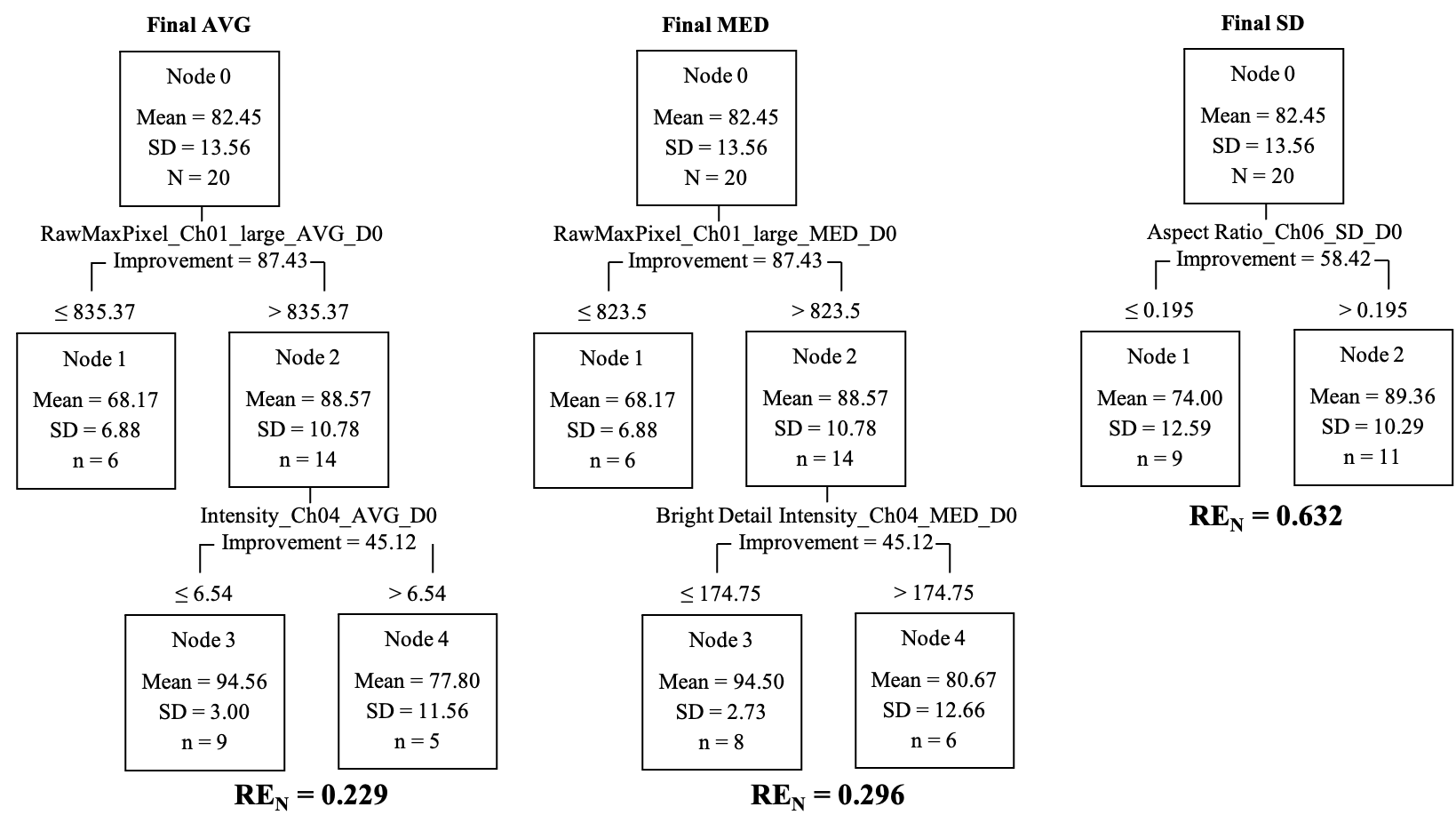


**Figure S4. Final regression trees (RT) of resilience using baseline EV features.** The average of two different signal strength features, the median of two distinct signal strength features, and the standard deviation of one shape feature able to discriminate resilience were included in the final RT models and used for subsequent statistical analyses. RE_N_ = normalized risk estimate.


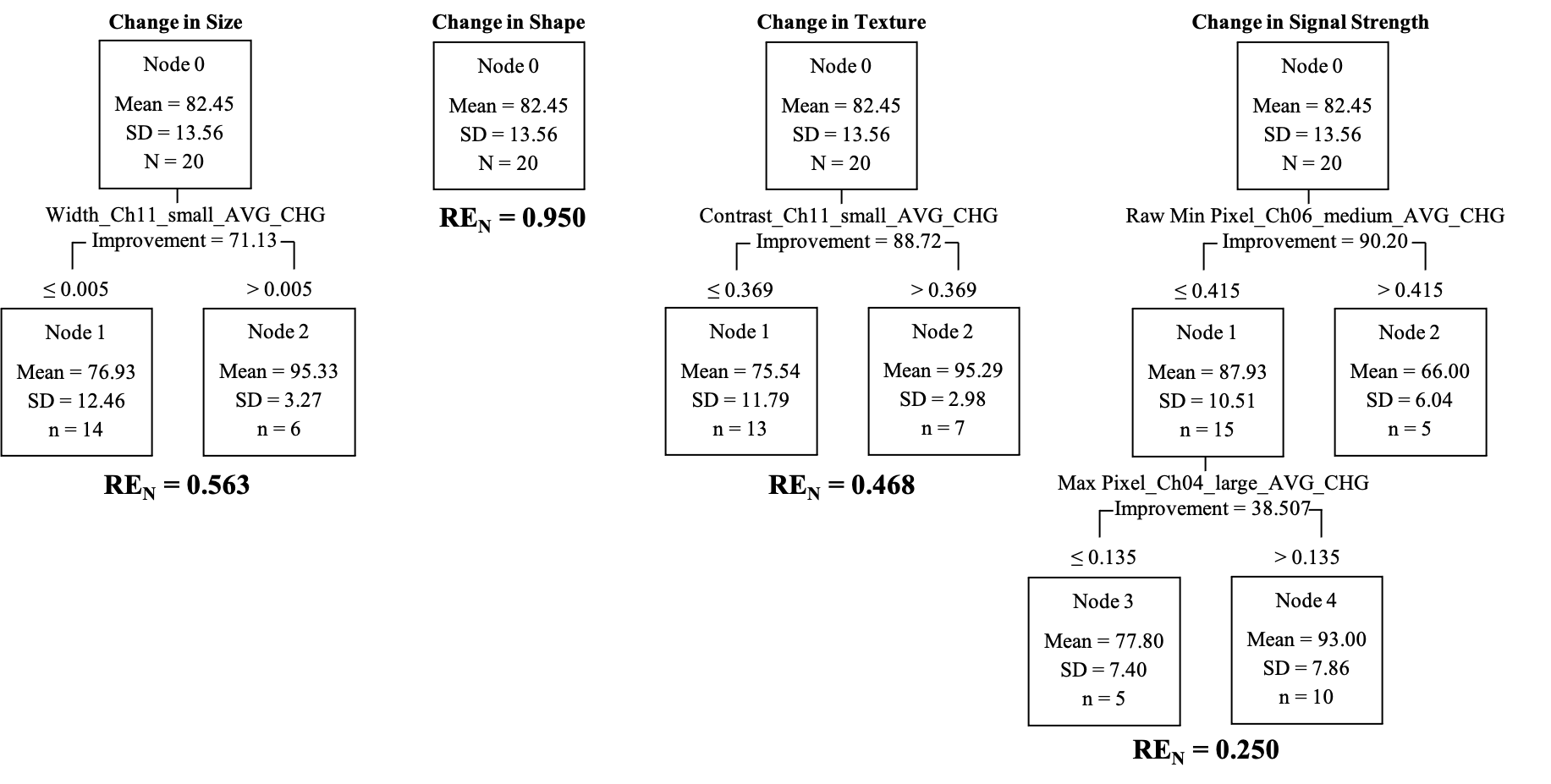


**Figure S5. Regression tree of resilience scores based on average (AVG) change in EV features from baseline to peak stress.** Average change in one feature within the size category, one feature of texture, and two features of signal strength were able to discriminate resilience; the decision tree for the shape category was unable to generate child nodes. RE_N_ = normalized risk estimate, CHG = change.


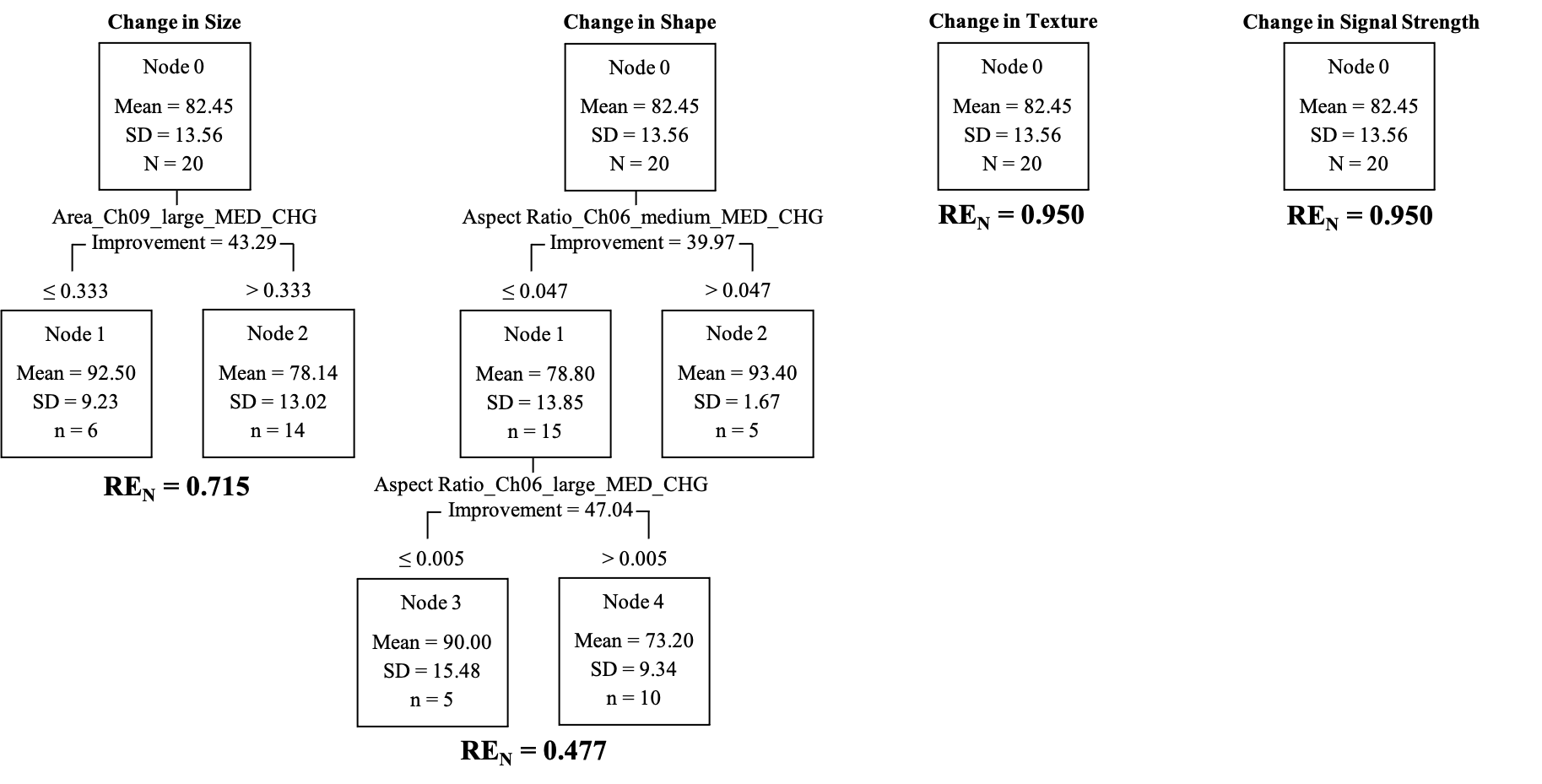


**Figure S6. Regression tree of resilience scores based on median (MED) change in EV features from baseline to peak stress.** The median change in one feature within the size category and two features of shape were able to discriminate resilience; the decision tree for the texture and signal strength categories were unable to generate child nodes. RE_N_ = normalized risk estimate, CHG = change.


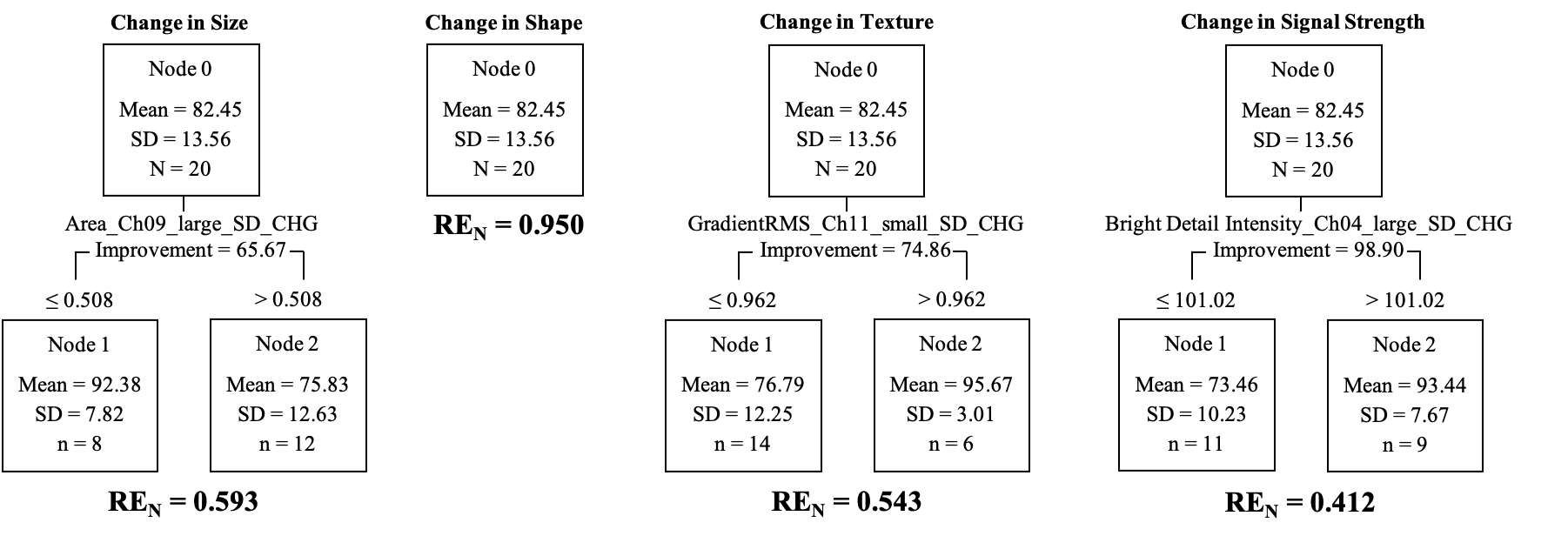


**Figure S7. Regression tree of resilience scores based on standard deviation (SD) change in EV features from baseline to peak stress.** The standard deviation of change, in other words, the variability of change, in one feature within the size category, one feature of texture, and one feature of signal strength were able to discriminate resilience; the decision tree for the shape category was unable to generate child nodes. RE_N_ = normalized risk estimate, CHG = change.


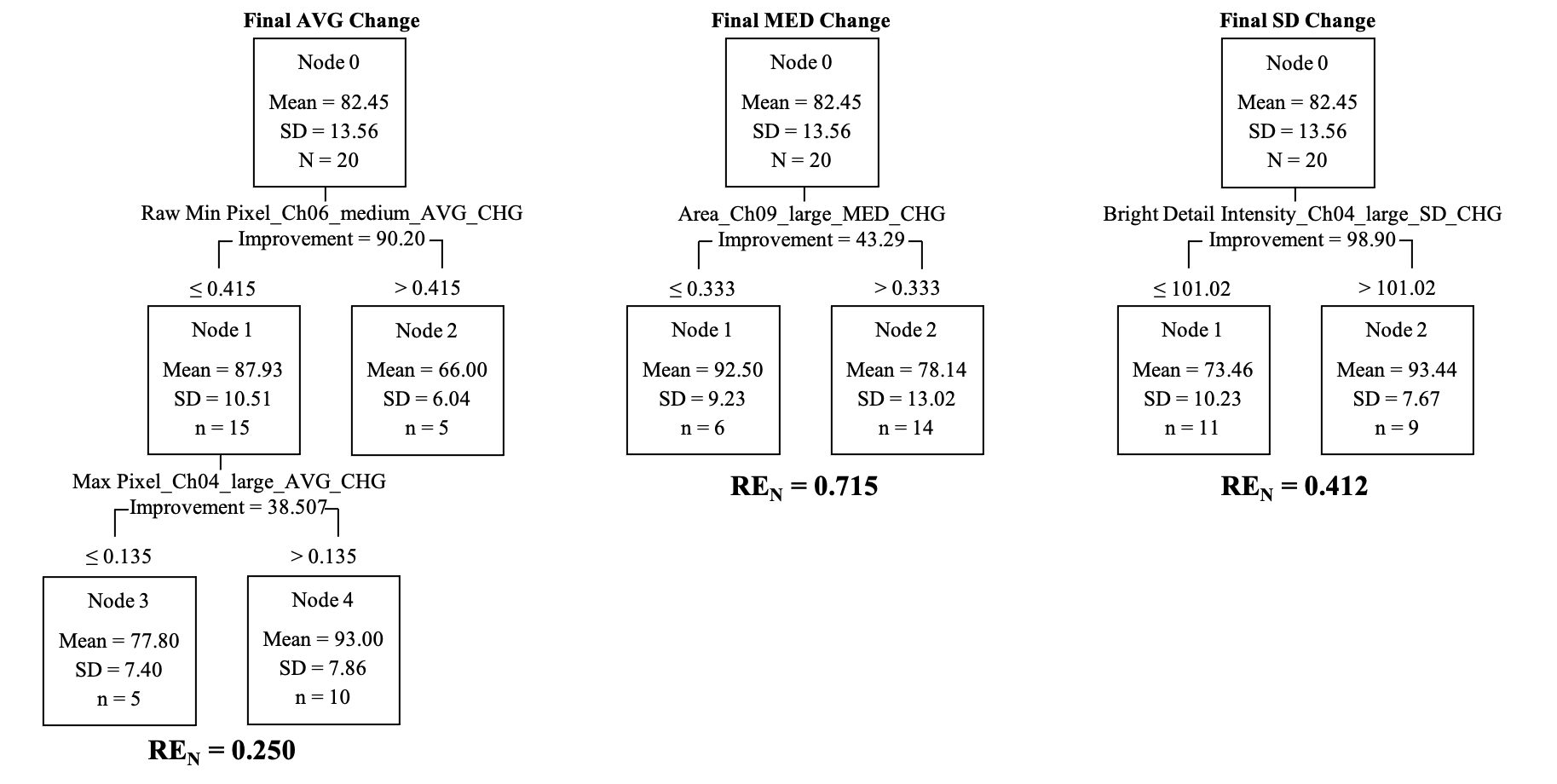


**Figure S8. Final regression trees (RT) of resilience based on changes in EV features from baseline to peak stress.** The average (AVG) change from baseline to peak stress in two different signal strength features, the median (MED) change of one signal strength features, and the change in the standard deviation (SD) of one signal feature able to discriminate resilience were included in the final RT models and used for subsequent statistical analyses. RE_N_ = normalized risk estimate. RE_N_ = normalized risk estimate.
